# The electrical restitution of the non-propagated cardiac ventricular action potential

**DOI:** 10.1007/s00424-023-02866-0

**Published:** 2023-10-03

**Authors:** Massimiliano Zaniboni

**Affiliations:** Department of Chemistry, Life Sciences and Environmental Sustainability, University of Parma (Italy), Parco Area Delle Scienze, 11/A, 43124 Parma, Italy

**Keywords:** Ventricular action potential, Cardiac electrical restitution, Ventricular repolarization, Cardiac arrhythmias, Restitution hypothesis, Antiarrhythmic drugs

## Abstract

Sudden changes in pacing cycle length are frequently associated with repolarization abnormalities initiating cardiac arrhythmias, and physiologists have long been interested in measuring the likelihood of these events before their manifestation. A marker of repolarization stability has been found in the electrical restitution (ER), the response of the ventricular action potential duration to a pre- or post-mature stimulation, graphically represented by the so-called ER curve. According to the restitution hypothesis (ERH), the slope of this curve provides a quantitative discrimination between stable repolarization and proneness to arrhythmias. ER has been studied at the body surface, whole organ, and tissue level, and ERH has soon become a key reference point in theoretical, clinical, and pharmacological studies concerning arrhythmia development, and, despite criticisms, it is still widely adopted. The ionic mechanism of ER and cellular applications of ERH are covered in the present review. The main criticism on ERH concerns its dependence from the way ER is measured. Over the years, in fact, several different experimental protocols have been established to measure ER, which are also described in this article. In reviewing the state-of-the art on cardiac cellular ER, I have introduced a notation specifying protocols and graphical representations, with the aim of unifying a sometime confusing nomenclature, and providing a physiological tool, better defined in its scope and limitations, to meet the growing expectations of clinical and pharmacological research.

## Introduction

Dynamics deals with forces that cause effects on motion. In the case of non-linear systems, changes in the effects are not proportional with changes of the cause and predicting the behavior of the former based on the knowledge of the latter becomes a hard task. I will review here the efforts of electrophysiologists in conceiving experimental measurements aiming to predict, in the case of the non-linear electrical dynamics of cardiac ventricular myocytes, what happens to their action potential (AP) duration (APD, the effect) when pacing cycle length (CL) changes (ΔCL, the cause). The measure of this cause-effect relationship goes under the name of electrical restitution (ER), whose features, summarized in different graphical representations (ER curves), will be reviewed in this article. AP repolarization is a key determinant of cardiac function, and changes in AP trajectory modulate refractoriness and excitation–contraction (EC) coupling, and, with those, also the cardiac propensity to develop arrhythmias [150]. The reason why ER has become so important for cardiac electrophysiology is that it captures relevant information concerning the stability of the ventricular AP repolarization under perturbation of the pacing rate. The so-called restitution hypothesis (ERH) predicts how repolarization changes due to pre- or post-mature stimuli are quenched in a few beats or amplified toward repolarization alternans depending on features of the ER curves [65, 105]. ERH, though limited to specific experimental conditions, has been the starting point for fruitful research in cardiac field, including cardiac memory, arrhythmogenesis, and development of antiarrhythmic drugs, which will be covered in this review. Over the years, cellular ER has been studied by many groups, either in vivo in electrically paced cardiac tissue or isolated myocytes, or in silico in numerical AP models, and many different experimental protocols have been adopted, together with a plethora of terms, indicating restitution properties. A synthetic summary of ER protocols will also be provided in this review.

Though the ultimate meaning of ER measurements is predicting repolarization stability and proneness to arrhythmias at the whole organ level, they are measured for example at multiple sites in hearts of patients [21, 25, 97, 98], restitution mechanism originates at the cell level. Intercellular gap junctional coupling and electrotonic interaction modulate cellular ER within the tissue and introduce a further level of complexity [147], which also comprehends the link between the slope of ER and AP conduction velocity [107, 108] and the interaction of ER properties between Purkinje fibers and ventricular tissue [16, 128]. All these supra-cellular issues will not be discussed here, where I will only focus on the non-propagated AP.

Similarly, from the technical point of view, the length of systole, diastole, and cardiac CL can be measured in different ways and at different levels of complexity, from the cellular, to the tissue, organ, and body surface level. Here I will focus exclusively on ER measured from markers of the cardiac cycle at the transmembrane level, thus including studies where *V*_m_ was measured by means of standard microelectrodes, patch clamp, monophasic action potentials (MAP), voltage sensitive dies, or by simulation with numerical AP models.

Finally, a brief note on the measure of APD is due here. AP duration is usually measured as the time between the positive peak of the first time-derivative of *V*_m_ (initial fast depolarization) and the time when *V*_m_ reaches a given value, most often − 50 or − 60 mV, or the time taken for *V*_m_ to reach 50%, or 90% of the full repolarization. To keep the notation simple, I will use the term APD without specifying the way duration was measured. The choice does not qualitatively modify results in most of the cases, particularly when referring, as in the present review, to the late repolarization phase. For details on how exactly APDs were measured, readers can refer to the cited references.

## From dynamic to standard restitution

To avoid using different names for the same phenomena and vice versa, it is useful to anticipate here some terms and procedures which will be discussed in further detail only later in the article, and particularly point out the difference between the steady-state rate dependence of APD and the standard restitution, respectively, called dynamic restitution (ER_dyn_) and S1–S2 restitution (ER_S1-S2_). The partition of the cardiac cycle CL into systole and diastole is chiefly, even though not exclusively, determined by mechanisms intrinsic to the cellular AP that initiates and controls the EC process, so that APD delimits the systolic contraction phase, whereas the rest of the cycle (diastolic interval, DI = CL − APD) corresponds to the relaxation phase of the heart chambers. As cardiac beating rate increases (CL decreases), to preserve enough diastolic time for the filling of heart chambers, APD, and the systole with it, become shorter. Let us consider pacing CL changing abruptly from one (CL_1_) to another (CL_2_ = CL_1_ + ΔCL) constant value and assume that both pacing trains last enough time for the corresponding APDs to reach their steady state. We can measure APD response to ΔCL by looking at the steady-state APD values, APD_1_ and APD_2_ respectively, or by focusing on the first APD at CL_2_. Iteratively applying different CL_2_ values with the first approach leads to measure the so-called steady-state APD rate dependence, or dynamic restitution, whereas the repetition of the second approach for different CL_2_ values provides the so-called standard restitution.

### The dynamic restitution (ER_dyn_)

**Fig. 1:**
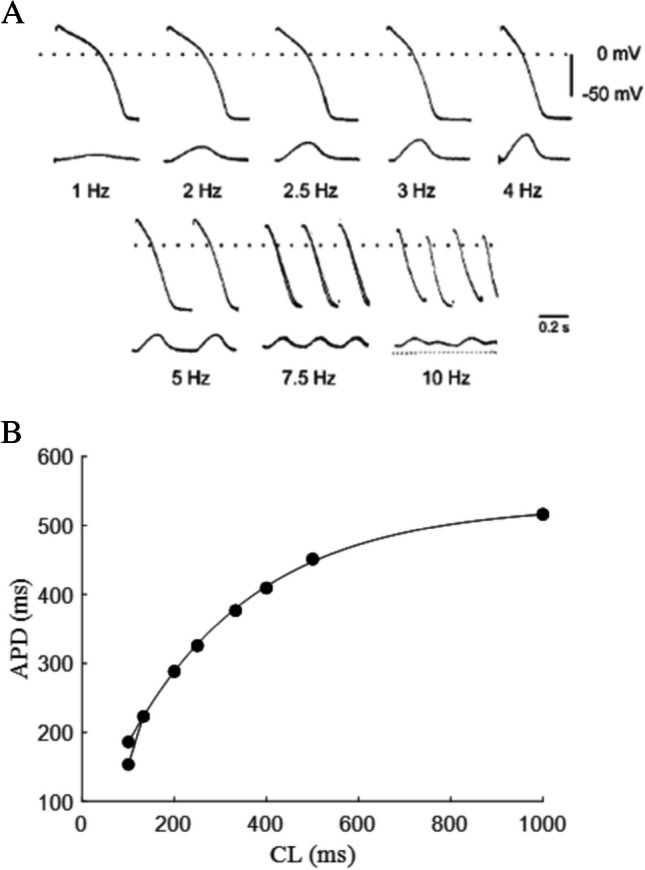
**Steady-state rate dependence of APD**. **A** APs (upper tracings) were recorded from guinea pig papillary muscle with standard microelectrodes, together with force of contraction (lower tracings). At each pacing frequency, time was allowed for the AP to reach the steady state (3–5 min) (figure from [125]. **B** APDs from panel **A**, measured at 90% of repolarization, are reported as a function of CL. Rate dependence curve is fitted with a mono-exponential. Note the bifurcation at CL = 100 ms (10 Hz) due to APD alternans at that pacing rate

The steady-state relationship between APD and pacing CL (or corresponding pacing DI) is represented by a monotonic function and can be obtained by pacing at constant CL for a given number of beats required for achieving steady state, and then progressively decreasing (or increasing) CL (Fig. 1). Since stationary conditions are achieved dynamically, it is also called dynamic restitution, or ER_dyn_, and the corresponding curve is unique for a given cell membrane. The APD vs CL and the APD vs DI representations of ER_dyn_ are different but contain the same information, i.e., they can be transformed into each other without further measurements. For this reason, though the term restitution is more often used for the latter, I will adopt it here also for the former. I note in passing that the term restitution is used improperly in both cases, as it has originally been formulated for the standard restitution (see next paragraph). Steady-state rate dependence of APD has long been known from physiologists. According to Galenus of Pergamon (129–216), the first who measured the duration of the cardiac cycle and of the systole was the Alexandrian physician Herophilos of Chalcedon in the fourth century B.C., by means of water clocks he developed [161]. Only after more than two millennia though, in 1920, the English physiologist Henry C. Bazett first described analytically the inverse relationship between cardiac beating rate and length of repolarization [10]. He did so by studying electrocardiograms,soon the observation was extended to microelectrode impaled preparations and allowed to establish what we now call rate dependence of APD [125]. ER_dyn_ will be treated in this review as a particular case of restitution.

### The standard restitution (ER_S1-S2_)

Right after a ΔCL, APD undergoes changes lasting a variable number of beats until it reaches back its steady-state value. The term restitution, from the latin verb restituere, giving back, refers originally to the return of the excitable system to its original stationary configuration after a single perturbation of the constant pacing CL. A number of protocols capture different aspects of the restitution dynamics with their different representations, or ER curves. One of these, the so-called standard restitution, or ER_S1-S2_, describes what happens to the steady-state APD waveform at a given constant basic CL (BCL, or S1) right after a single stimulus at a time S2, delayed or anticipated with respect to S1, is introduced. As for the steady-state ER_dyn_, ER_S1-S2_ curve is most of the times graphically described by a monotonic function which, unlike ER_dyn_, is not unique for a given membrane, but rather defined for any given BCL.

The development of the ER concept and the measure of ER curves are the object of the next two chapters.

## Brief history and main features of cardiac cellular ER

### In the beginning was the post-extrasystolic

**Fig. 2:**
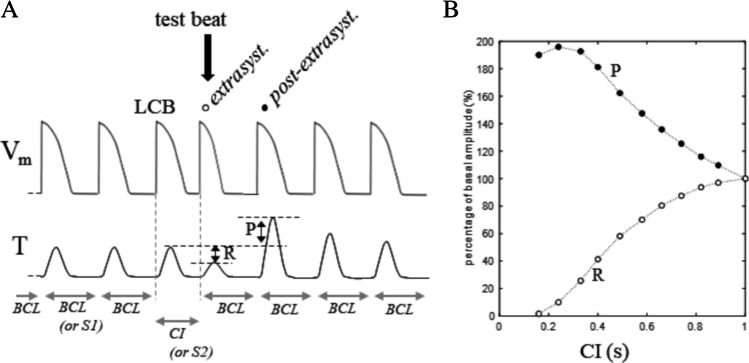
**Restitution and potentiation**. **A** Schematic representation of the experiment of Hoffmann and coworkers [63]. During constant BCL pacing, a single pre/post-mature stimulus is introduced (black arrow) at a given coupling interval (CI) after the last conditioning beat (LCB). Transmembrane potential (V_m_) and isometric tension (T) are reported. **B** By adopting the same protocol, Kruta and Braveny studied the percent changes of T developed by the extrasystoolic (restitution R) and post-extrasystolic (potentiation P) beats when CI was made varying (results from [84]

It has long been known that a single premature ventricular excitation during the late systole can initiate ventricular fibrillation (VF) [169], which is one of the reasons why electrophysiologists are interested in understanding and predicting the repolarization dynamics of an AP elicited under such conditions. Early studies on the effect of introducing extra-systolic beats during a pacing protocol at constant BCL where focused more on the post-extra-systolic beat than on the extra-systolic one [63]. In their cat papillary muscles work on the effect of a single premature beat, delivered at a time (coupling interval CI) pre- or post-mature with respect to BCL, Hoffmann described in fact the potentiation, i.e., the increase in the tension developed by the post-extra-systolic beat, with respect to the control, and its recovery back to control over few beats (Fig. 2A). They did record transmembrane APs with microelectrodes, without noting though significant changes in APD throughout potentiation. The difference between CI dependence of extra-systolic and post-extra-systolic beats (respectively restitution and potentiation) was analyzed in detail by Kruta and Braveny [84, 85]. In their works, the term restitution is first introduced, though still referred to the case of contractility (Fig. 2B).

### CI as independent variable

**Fig. 3:**
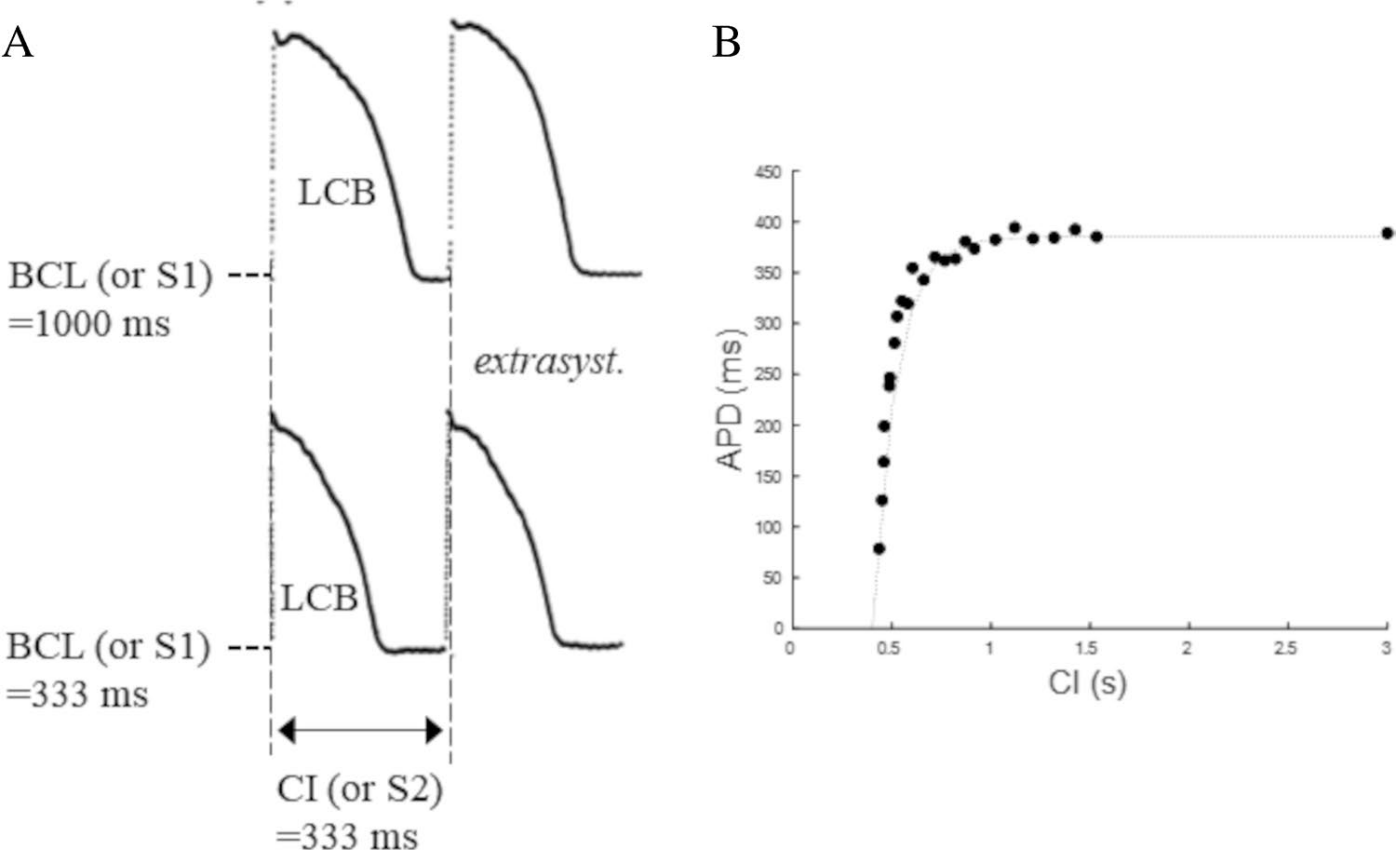
**The ER**_**S1-S2**_ **curve**. **A** top: after the LCB of a constant pacing train at BCL = 1000 ms (S1), a premature stimulus (S2, or CI = 333 ms) was applied and the test APD recorded. Bottom: an identical CI (BCL = 333 ms) was applied after a different BCL = 333 ms. Note that, since CI = BCL in this case, the test APD remains at its steady state and is identical to LCB (from [96]. **B** By applying the same S1–S2 pacing protocol, Boyett and Jewell measured the ER representation with the test APD as a function of CI (data from [17]

**Fig. 4:**
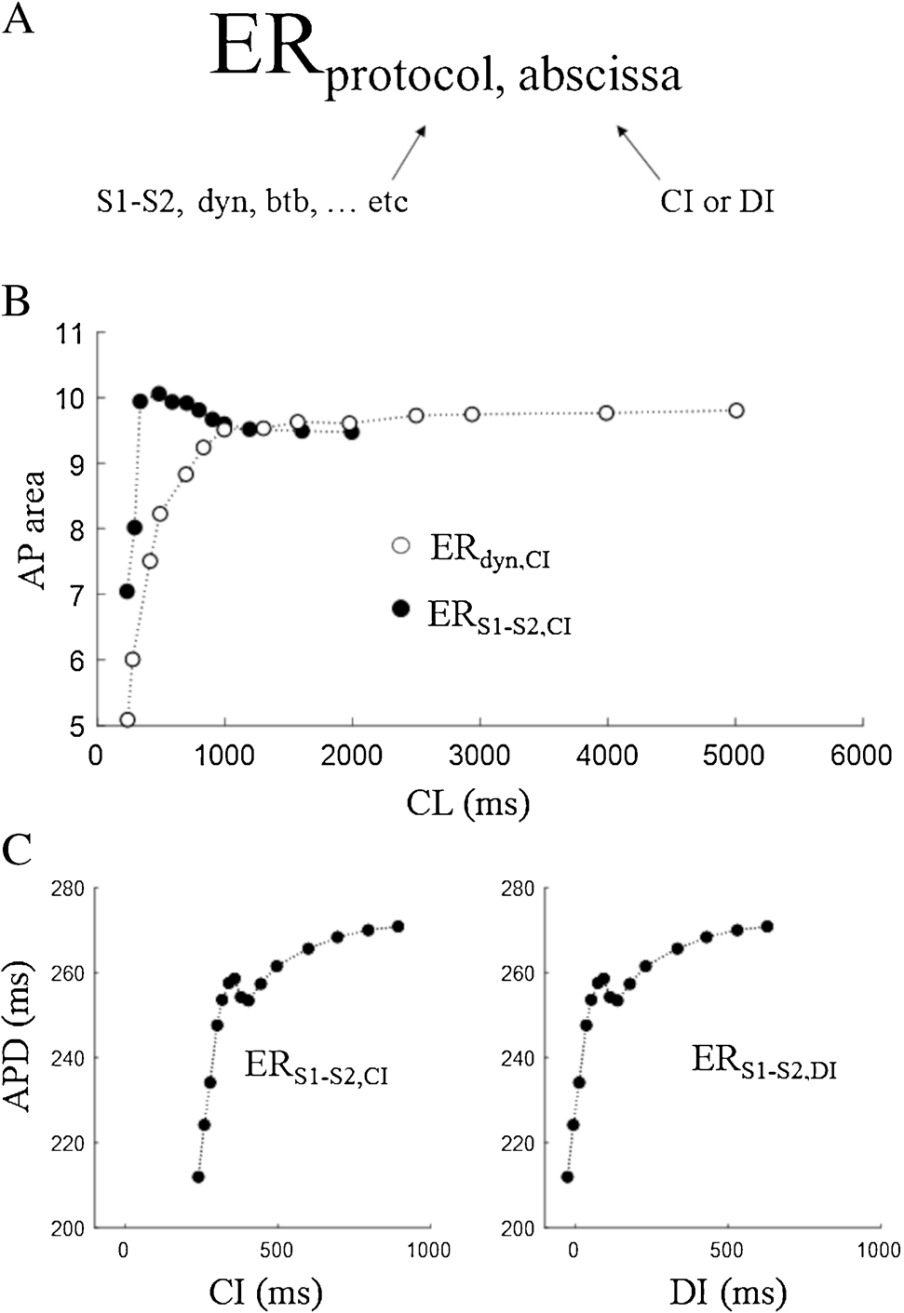
**Notation**. **A** The different types of ER will be identified here with two subscripts, the first referring to the protocol used to measure it, the second to the beating parameter reported in the x-axis of the corresponding ER curve. For example, ER_dyn,DI_ indicates electrical restitution measured with the dynamic protocol, and reported as APD versus DI. **B** An example of steady-state dog ventricular APD rate dependence (Er_dyn,CI_) is reported in figure with empty dots, superimposed to the ER curve measured with S1–S2 protocol (BCL = 2 s) (black dots) (data from [95]. Note that the x label CL represents CIs in the case of ER_S1-S2_, and BCLs in the case of ER_dyn_. **C**, left Example recorded in cat papillary muscle of restitution measured with S1–S2 protocol (BCL = 600 ms) and represented as APD vs CI. **C**, right Same data as in left panel, reported as APD vs DI (data from Franz et al. 1988)

Perhaps, the first report of what we now call ER_S1-S2_ is that of Moore and colleagues [96] on microelectrode recorded APDs from dog false tendons and ventricular muscle. They studied the APD dependence from an extra-stimulus (CI, or S2), equally anticipated with respect to the BCL of conditioning pacing (S1) (Fig. 3A), the disparity of this property between specialized conduction tissue and ventricular muscle and the relevance in the genesis of VF. That of Moore will become the standard procedure to measure ER_S1-S2_, also called S1–S2 protocol. Results like those of Fig. 3A have then been represented like two-dimensional plots with CI (also called test interval) in abscissa and test APD (or its percent changes) in the ordinate axis, under the name of ER_S1-S2_ curve (Fig. 3B). For the sake of clarity, I will call this representation the ER_S1-S2,CI_, the second subscript referring to the independent variable CI (see scheme in Fig. 4A). This curve, increasing exponentially in its early phase and then reaching a plateau for longer CIs (Fig. 3B), reflects the time course with which membrane ionic conductances underlying the last conditioning beat (LCB) return to their pre-stimulus value within the CI [17]. In early studies, repolarization changes were frequently measured through the AP area, instead of APD, like in the work of Miller and colleagues [95]. They measured the effect of premature beats in constantly paced canine ventricular muscle and Purkinje fibers. Starting from different BCLs (S1, they suddenly switched to a common shorter constant pacing CL (CI or S2 and described the APD adaptation, i.e., the time course of the AP area in reaching the common steady-state value,this will later be called the constant BCL protocol (ER_CB_, see paragraph 4.3). In the same study, they provided a representation of the ER_S1-S2,CI_ curve for the AP area, reported in Fig. 4B (filled dots), superimposed to the ER_dyn,CI_ (empty dots), in the case of ventricular tissue. Notably, and unlike most of the experimental findings, the curve does not grow monotonically here; it does in Purkinje fibers (not shown), but it rather shows a more complex form. Cases in which the initial part of the ER curve is triphasic have been shown, first by Bass in cat papillary muscle [8, 9] and then, for example, by Franz and colleagues in human heart in situ [41] (Fig. 4C, left) and will be discussed later in this article.

### DI as independent variable

The ER representation shown in the left panel of Fig. 4C can be given in a different form by reporting in abscissa the DI, instead of the CL, preceding the test beat, thus, according to our notation, an ER_S1-S2,DI_ curve (right panel). Since DI depends linearly on CI, the two representations are identical except for a translation along the x axis. The ER_S1-S2,DI_ though, for reasons that will be discussed below, has soon become the most adopted representation for restitution.

### Comparison of ER curves

**Fig. 5:**
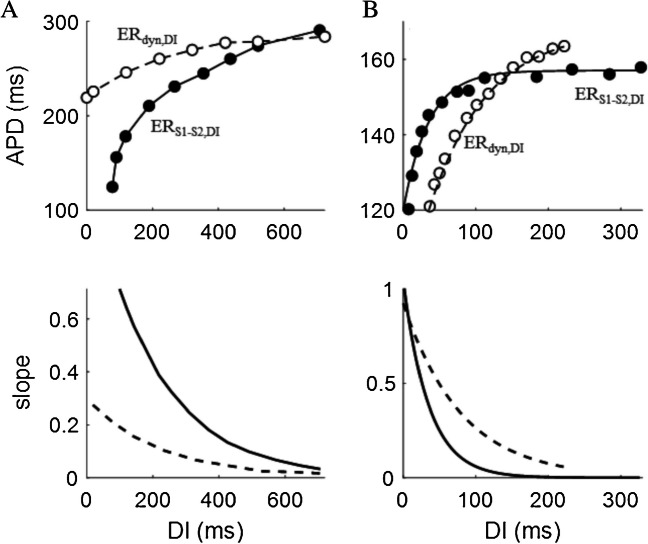
**ER**_**S1-S2,DI**_ **vs Er**_**dyn,DI**_. **A**, top Dynamic (ER_dyn,DI_, empty dots) and standard (ER_S1-S2,DI_, filled dots) restitution optically measured in canine left ventricular wedge. **A**, bottom Slopes of the above curves (dotted line for ER_dyn,DI_ and solid line for ER_S1-S2,DI_) (data from [188]. **B** Same for curves measured with MAP recordings in left ventricular epicardium of Langendorff-perfused guinea pig hearts by Soltysinska and colleagues [140]

**Fig. 6:**
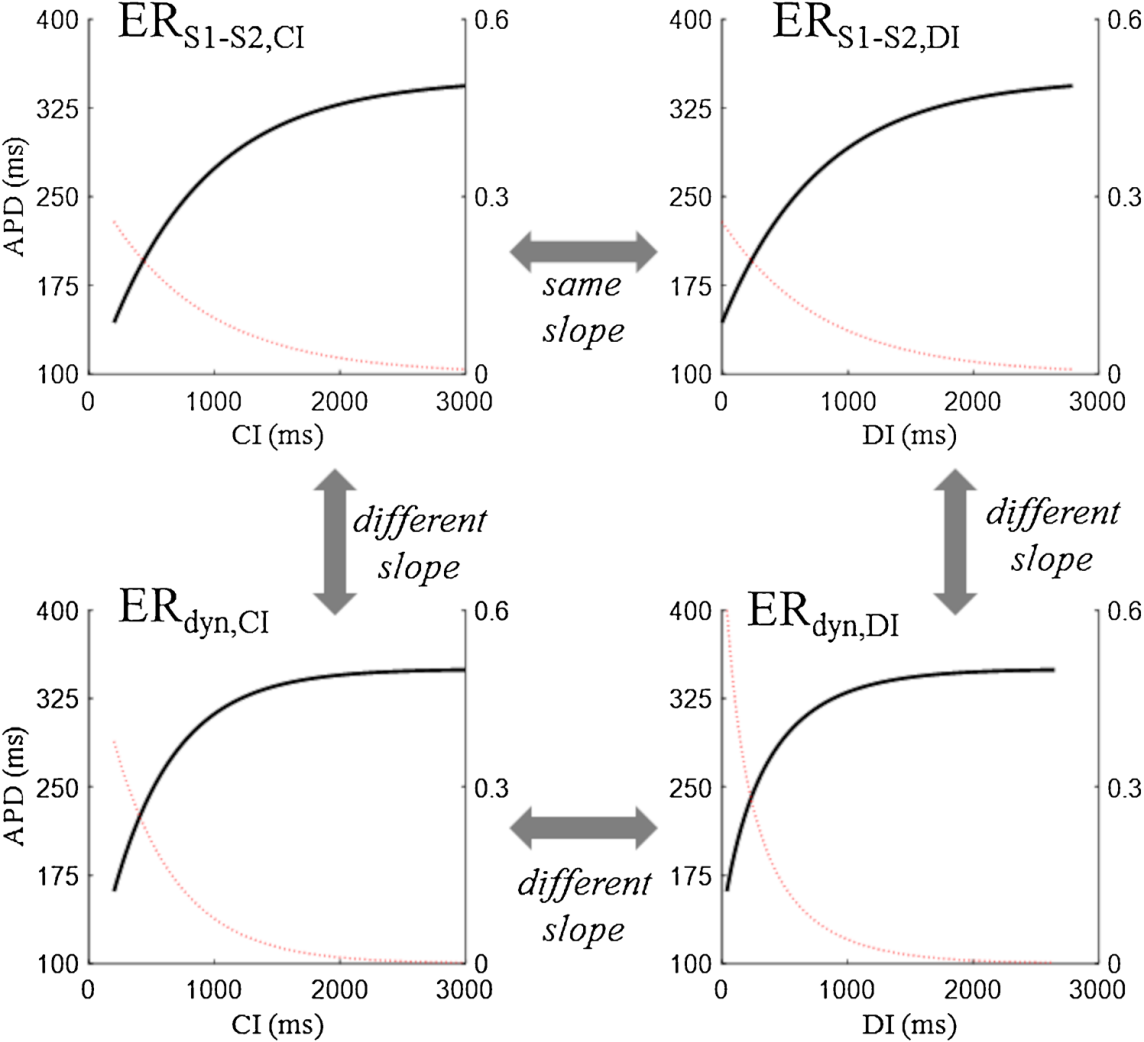
**Comparison of ER formulations**. In all panels, representative ER curves (black line, left y axis) and their slopes (red line, right y axis) are reported. (top) A generic ER_S1-S2,CI_ has the same shape of the corresponding ER_S1-S2,DI_ measured for the same cell at the same BCL. (bottom) The steady-state ER_dyn,CI_ is unique for the same cell, different from the instantaneous ER_S1-S2,CI_, and also different from the corresponding ER_dyn,DI_

Both ER_S1-S2,DI_ and ER_dyn,DI_ are frequently adopted for studying and representing restitution. In Fig. 5A, an example of the two, optically measured by Zhang and colleagues in wedges of dog left ventricle, is reported. Note the different shape and APD range of the two; in this case, slope and maximum slope are much higher in ER_S1-S2,DI_ [188]. The relationship between slopes of dynamic and standard curves is reversed in the case reported in Fig. 5B. In the scheme of Fig. 6, the four ER representations described so far are compared. ER_S1-S2,CI_ and ER_S1-S2,DI_ are essentially the same curve, only shifted horizontally (same shape and slope). Since they represent steady state and instantaneous responses respectively, ER_dyn_ and ER_S1-S2_ are in general different in shape and slope. Also, ER_dyn,DI_ is different with respect to ER_dyn,CI_ by definition, since the abscissa DI depends non-linearly from CI. At the steady-state conditions, in fact, DI = CL – APD and APD, according to ER_dyn,CI_, depends non-linearly on CL.

### BCL dependence of ER_S1-S2_

**Fig. 7:**
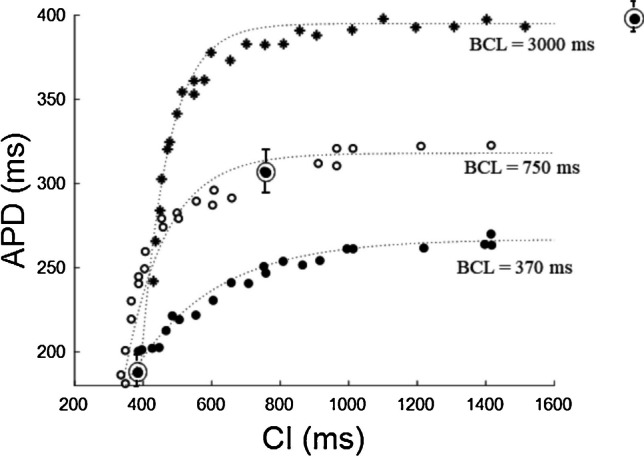
**BCL dependence of the ER**_**S1-S2**_ **curve**. ER_S1-S2_ curves depends, by definition, on the BCL of the conditioning pacing. In figure, an example where differently delayed S2 stimuli were delivered, in turn, after a conditioning training at 3 different values (reported in figure) of BCL (3000, 750, and 370 ms) in superficial cells of cat papillary muscle. Data are fitted with mono-exponentials with time constants *τ* = 90, 170, and 300 ms respectively (data from Boyett and Jewel 1978)

Being the ER_S1-S2_ curve defined for a given conditioning pacing rate, it is not surprising that its shape changes according to the BCL value. As Boyett and Jewell first showed in their fundamental work on microelectrode impaled cat papillary muscle [18], decreasing BCL values not only shifts the ER_S1-S2,CI_ curve downward but also changes its shape and APD range (Fig. 7). In the example in figure, with decreasing BCL, the time constants of the mono-exponential ER curve fittings dramatically increase. Of course, the same BCL dependence can be found in ER_S1-S2,DI_ representations [109, 110, 145].

### Species and spatial specificity

**Fig. 8:**
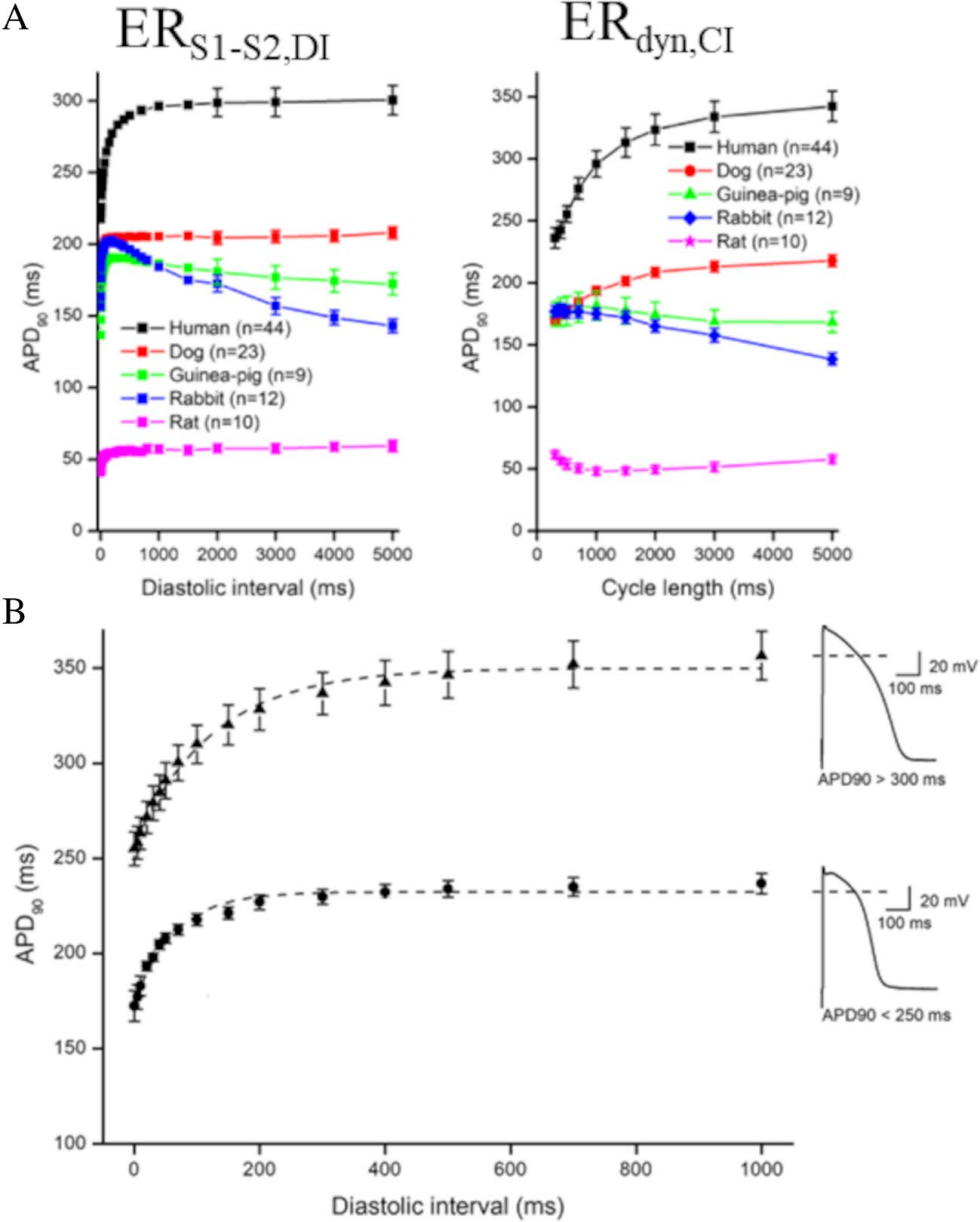
**Species and spatial heterogeneity of ER**. **A** Differences in ER_S1-S2,DI_ and in steady-state ER_dyn,CI_, documented by Arpadffy-Lovas in microelectrode impaled tissue preparations from different species. **B** Differences in ER_S1-S2,DI_ depending on the APD measured at CL = 1000 ms (insets) in the same species (human). ER_S1-S2,DI_ curves were fitted by mono-exponentials. Time constants of ER_S1-S2,DI_ curves averaged 63.9 ± 6.0 ms for APD < 250 ms (*n* = 10, filled squares), and 125.5 ± 9.1 for APD > 300 ms (*n* = 17, filled triangles) (data and figure from Arpadffy-Lovas et al. 2020)

Ventricular APs differ in waveform, duration, and dynamics among species and, within the same species, between the right and left ventricle and among different ventricular regions [2, 51, 57, 73, 135]. Electrical restitution is subject to analogous heterogeneity [19, 101, 143]. An extreme case is that of the rat ventricle, where, due to its peculiar intracellular calcium dynamics, the slopes of ER_S1-S2_ and ER_dyn_ curves are negative [101]. Differences between epicardial and endocardial ER_dyn_ and ER_S1-S2_ have been shown by Litovsky in dog right ventricle and attributed to the different expression of potassium current I_TO_ in the two ventricular regions [91]. Arpadffy-Lovas and colleagues have recently compared ventricular ER_S1-S2,DI_ and ER_dyn,CI_ curves measured in human and other mammalian species (Fig. 8A) [3] Besides being shifted vertically according to the species-specific steady-state APD, the ER curves show quite different shapes. Notably, even in the same species (human), they show different behavior depending on the heterogeneously distributed intrinsic AP dynamics, analyzed by the authors in the case of steady-state duration (Fig. 8B) and in that of the amplitude of early AP repolarization (not shown).

### Restitution of EC-coupling markers

The above ER_S1-S2_ and ER_dyn_ protocols can be applied not only to APD, but to any of the variables involved in the cardiac EC coupling. Thus, ER curves for the amplitude of ionic currents underlying the AP [68, 152, 119] or for any membrane electrogenic process active during the cardiac cycle, for the amplitude, time to peak or rate of relaxation of the calcium transient, and for cell contraction, have been measured and studied [45, 138].

### The analytic form of ER

ER curves in the forms described in Fig. 6 are usually fitted with mono- or, more frequently, bi-exponential functions [17, 36, 71, 93], whose time constants are used to quantify the slope of the curve in its early and late phases (shorter and longer CIs or DIs) and compare them in different substrates. For an overall quantification of the slope of the curve, the maximum slope reached (S_max_) is also used.

## The many restitutions

The steady-state ER_dyn_ and the standard ER_S1-S2_ are the two most frequently used approaches to study restitution dynamics of cardiac cells. Over the years, additional methods have been established to access ER properties, each one with a corresponding graphical representation [20, 36, 56, 116, 149]. I will list them here and briefly describe the experimental protocols for their measure. Some of these have been introduced above,some will be discussed in further detail in the following chapters. The notation introduced in Figure 4A will be used for each protocol.

### The standard ER curve (ER_S1-S2_)

APs are electrically elicited at a constant conditioning BCL (S1) until they reach steady-state configuration. After the LCB, a stimulus is delivered at variable CIs (S2, or test intervals), which elicits a further AP (extra-systolic or test beat). The ER curve represents the test APD versus CI (ER_S1-S2,CI_), or versus the corresponding DI = CI − APD_BCL_ (ER_S1-S2,DI_) [17, 36, 42]. The ER_S1-S2_ curve is not unique for a cell but defined for a given BCL and in general different for any BCL (Fig. 7). S1-S2-S2 protocols have also been used, where two test APs are elicited at the same CI (S2) after the LCB [78].

### The dynamic ER curve (ER_dyn_)

BCL is kept constant for a fixed number of beats (enough to reach steady state, typically 50), the last APD measured, and pacing resumed for the same number of beats at a new shorter constant BCL. The ER_dyn_ curve is unique for a given cell and different, in general, from the ER_S1-S2_ curve, either shallower [79] or steeper [17, 69, 79] (see Fig. 5).

### The constant BCL ER curve (ER_CB_)

BCL is kept constant until AP reaches steady state and then suddenly shortened to a new constant value. The transient response of APD as it relaxes to the new steady state, reported as APD vs the preceding DI for each beat, provides the ER_CB,DI_ curve, also called constant BCL restitution [46, 116, 148].

### The AP-clamp derived ER curve of ionic fluxes

**Fig. 9:**
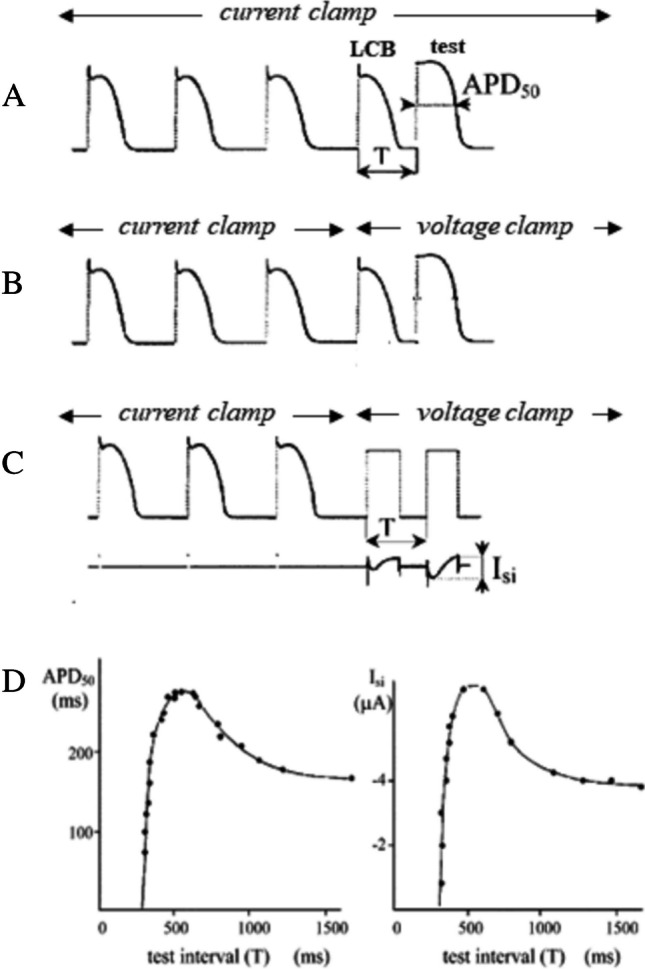
**AP-clamp derived ER curve for ionic currents**. Ideally, LCB and test APs should be recorded during a S1–S2 protocol (**A**) and applied as voltage clamp pulses after conditioning training (**B**). In a much easier version, two identical voltage clamp square pulses are used instead under calcium current I_si_ blockade, and the ionic current difference between the test beat and LCB used to describe the ER curve of I_si_ (right **D** panel). ER_S1-S2_ of APD is also reported on the left (figure from [138]

It is a modified S1-S2 protocol combining current and voltage clamp. AP waveforms of the LCB and of the test beat at a given CI are recorded and used to voltage clamp the cell membrane after conditioning pacing at constant BCL in control conditions, and under selective block of a given ionic current or electrogenic transporter (Fig. 9). The integral of the current response provides the charge crossing the membrane during LCB and test APs. The difference of the two as a function of CI represents the ER curve of the charge transferred by the blocked current [138]. Not to be confounded with the ER curve of ionic currents measured with constant voltage clamp pulses, which provides their general restitution dynamics independently from the specific AP dynamics, this type of protocol provides a solid theoretical ground for deeper investigation into both numerical and experimental research in cardiac restitution. Technically difficult to apply in experimental conditions, it allows peel onion type AP-clamp dissection [7] of all ion fluxes contributing to ER. A deeper analysis of this protocol cannot fit the present review.

### The ER surface

**Fig. 10:**
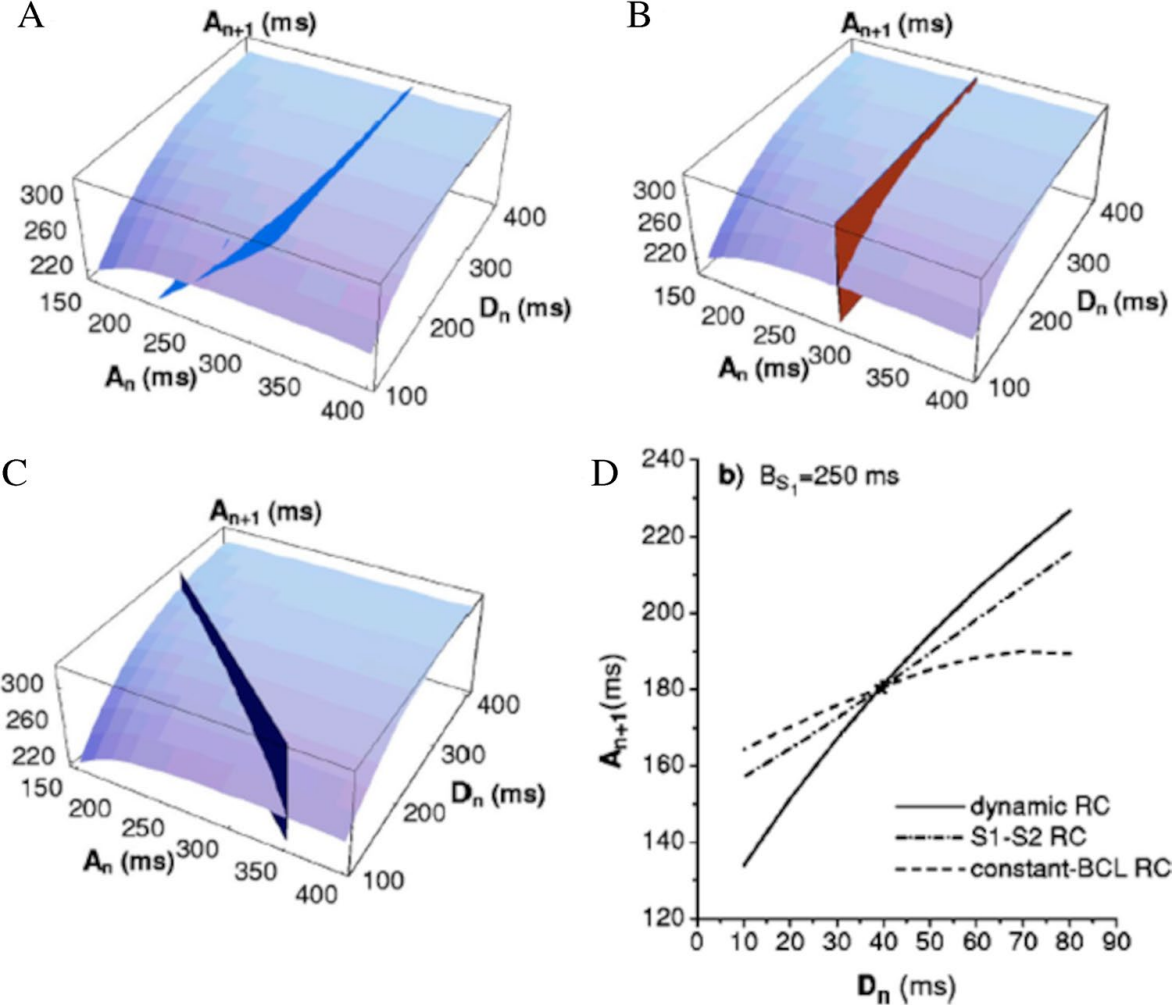
**The ER surface and the portrait**. Restitution dynamics when pacing CL suddenly changes between two constant values (BCL_1_ and BCL_2_) can be though off as a function mapping the LCB at BCL_1_ (*A*_n_) and the following DI (*D*_n_) into a surface describing all the possible states of the test APD (*A*_n+1_). ER_dyn,DI_, ER_S1-S2,DI_, and ER_CB,DI_ can be derived as the intersections of this surface with the three flat surfaces *A*_n+1_ = *A*_n_ (panel **A**), *A*_n_ = *A*_LCB_ (panel **B**), and *A*_n_ + *D*_n_ = BCL_2_ (panel **C**) respectively. The restitution portrait results from the projection of these intersecting curves on the (*A*_n+1_, *D*_n_) plane and is therefore the collection of the three ER curves (panel **D**). These tend to coincide for large values of BCL_1_ (low pacing rate, not shown). At high pacing rate, though, they rotate with respect to each other around the point representing steady-state DI and APD at BCL_1_ (figures from [148]

When a constant pacing BCL_1_ is suddenly increased (or decreased) to a new constant value BCL_2_, restitution dynamics can ideally be summarized with a function that maps the APD of the LCB at BCL_1_ (APD_n_) and the following DI (DI_n_) into a surface that describes all the possible states of the test APD (APD_n+1_) [148] (Fig. 10). This surface, as Tolkacheva elegantly shows, contains all at once the information of protocols described at points 4.1, 4.2, and 4.3. In particular, the ER_S1-S2,DI_ curve results as the intersection of this surface with the plan APD_n_ = APD_LCB_, the ER_dyn,DI_ curve from the intersection with the APD_n+1_ = APD_n_, and the ER_CB,DI_ curve from the intersection with the plan APD_n_ + DI_n_ = BCL_2_ (panels A, B, and C of Fig. 10). To note, the surface is only defined for a given BCL_1_, and, similarly to the above protocols, only describes the restitution dynamics of a stationary APD.

### The ER portrait

When, in the above three-dimensional representation, the 3 intersecting curves are projected on the (APD_n+1_, DI_n_) plan, the result is what Kalb and colleagues have called restitution portrait (Fig. 10D), which compactly summarizes all the ER properties described above [70, 148, 149, 188]. Kalb suggests a straightforward experimental protocol that allows constructing the portrait [70].

### The beat-to-beat ER curve (ER_btb_)

**Fig. 11:**
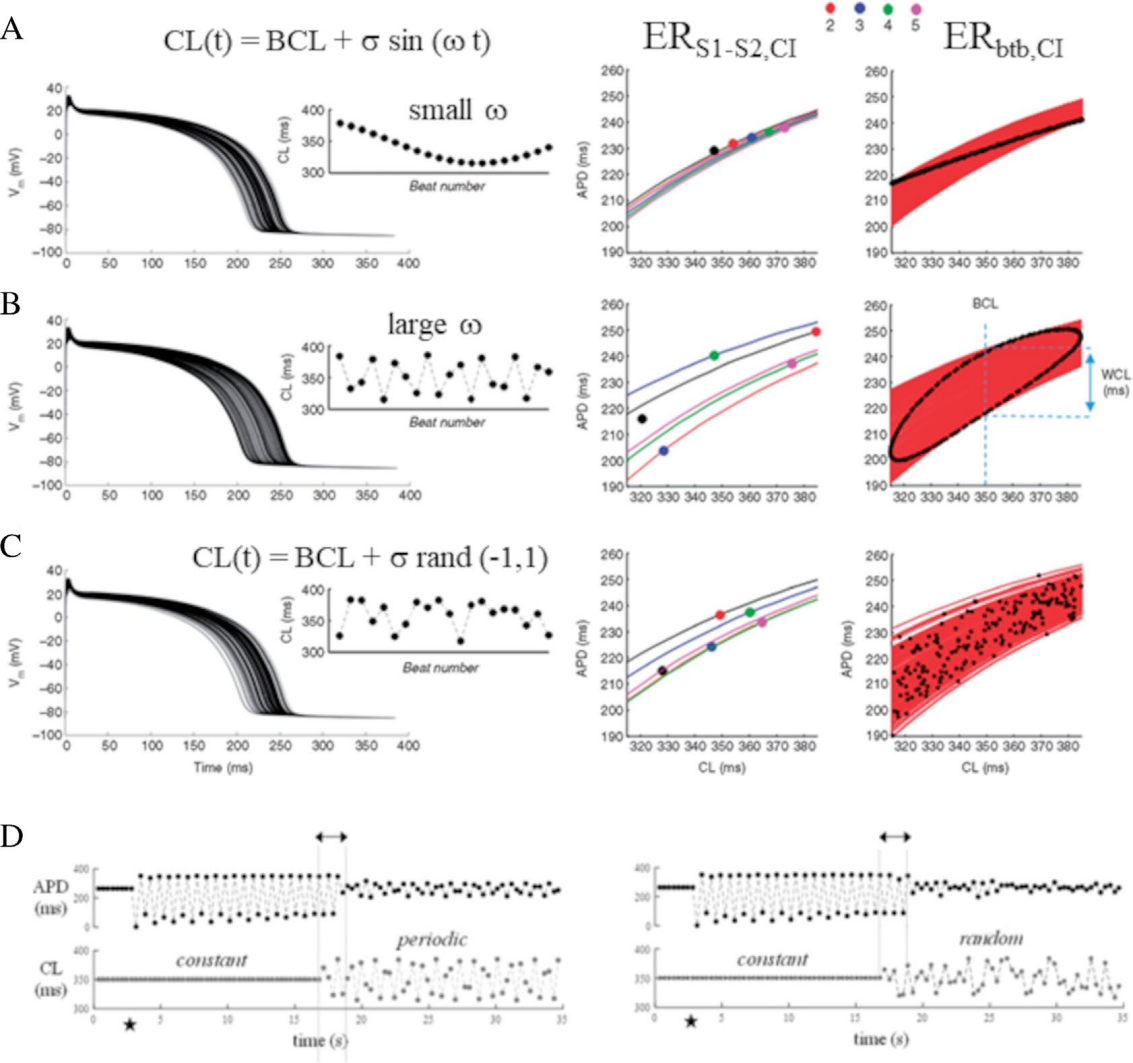
**ER**_**btb**_ **and family of ER**_**S1-S2**_ **curves**. The ten Tusscher human ventricular AP model [147] was paced with CL sequences varying **A** sinusoidally with small angular frequency (*ω* = 0.4), **B** sinusoidally with large angular frequency (*ω* = 2.4), and **C** randomly. The average CL (BCL) and its variability window (*σ*) were the same in the three instances. One hundred APs of each sequence are reported, superimposed, in the first column. Insets show detail of APD sequences. The second column reports APD_n_,CL_n-1_ values of five consecutive beats (see color code) and their corresponding ER_S1-S2_ curves. In the third column, all ER_S1-S2_ curves measured for each of the 100 beats are reported in red. The collection of the APD_n_,CL_n-1_ values for all beats is reported as black dots and form the corresponding ER_btb_ representations (data and figure from [185]. **D**, left The AP model was initially paced with a constant CL = 350 ms (lower panel). The initial steady-state APD (upper panel) was turned into APD alternans by switching off the current stimulus for one single beat (black star). APD alternans was removed by passing to pacing CL oscillating sinusoidally around 350 ms within a ± 50 ms range and at an angular frequency *ω* = 2.4. To note, *ω* values lower than 2.4 failed to stop alternans. **D**, right Same as on the left but with variable pacing following a random law

All the above protocols measure restitution of a steady-state AP waveform. ER dynamics, though, can be assessed also in non-stationary pacing conditions by graphically reporting, under beat-to-beat variable-CL pacing, each APD as a function of the preceding CL (or DI) (Fig. 11A–C, third column). CL can be made varying, within a given range, according to a sinusoidal, random, saw-tooth, or any temporal law [1, 13, 34, 35, 55, 90, 123, 178, 180, 183–185]. A pacing procedure has also been established by Wu and Patwardhan to control, on a beat-to-beat basis, the diastolic interval DI instead of CL, thus enabling to make DI following any programmed time law and providing the corresponding ER representations [55, 69, 173, 174]. The ER_btb_ has been adopted in experimental and numerical studies. I have measured it with my collaborators in rat ventricular myocytes [180], and in numerical models of ventricular AP, by means of constantly, sinusoidally, and randomly varying CL sequences [180, 183, 184, 186]. This type of restitution is used extensively for representing QT versus RR in electrocardiographic recordings.

### Family of ER_S1-S2_ curves

If, at any time during a beat-to-beat variable pacing, we want to answer the question “what will be the APD of the next beat for whatever following CI?”, the answer is the ER_S1-S2_-type curve for that beat. Applying different coupling intervals (S2) to the same beat of a variable-S1 stimulation can result extremely difficult in vivo but can easily be achieved in numerical simulations with AP models, where an ER_S1-S2_ curve can be derived for every beat of an AP sequence (Fig. 11A–C, second column), with significance for APD memory and stability (see paragraph 7.2).

Although either ER_S1-S2_, ER_dyn_, ER_CB_, and ER_btb_ provide useful descriptions of AP repolarization dynamics, it is not always possible to measure them in the different experimental conditions. In a clinical setting, for example, ER properties are usually assessed by the S1–S2 protocol [102, 103, 133, 144], as dynamic pacing may provoke tachycardia, thus raising safety concerns [109, 110].

## The restitution hypothesis

Stability is the property of a system, when perturbed from a condition of equilibrium, to develop forces that restore its original condition; thus, ER_S1-S2_, as it is formulated, is a measure of repolarization stability when constant pacing rate is perturbed. If a given ΔCL produces a small APD variation, i.e., small ER_S1-S2_ slope, it means that APD tends to be independent from CL changes (more stable). If the same ΔCL leads to a large ΔAPD (greater slope), then APD is more sensitive to changes in pacing rate (less stable). In this sense, the slope of ER_S1-S2_, either ER_S1-S2,CI_ or ER_S1-S2,DI_ is, already qualitatively, a marker of APD stability.

### The hypothesis

**Fig. 12:**
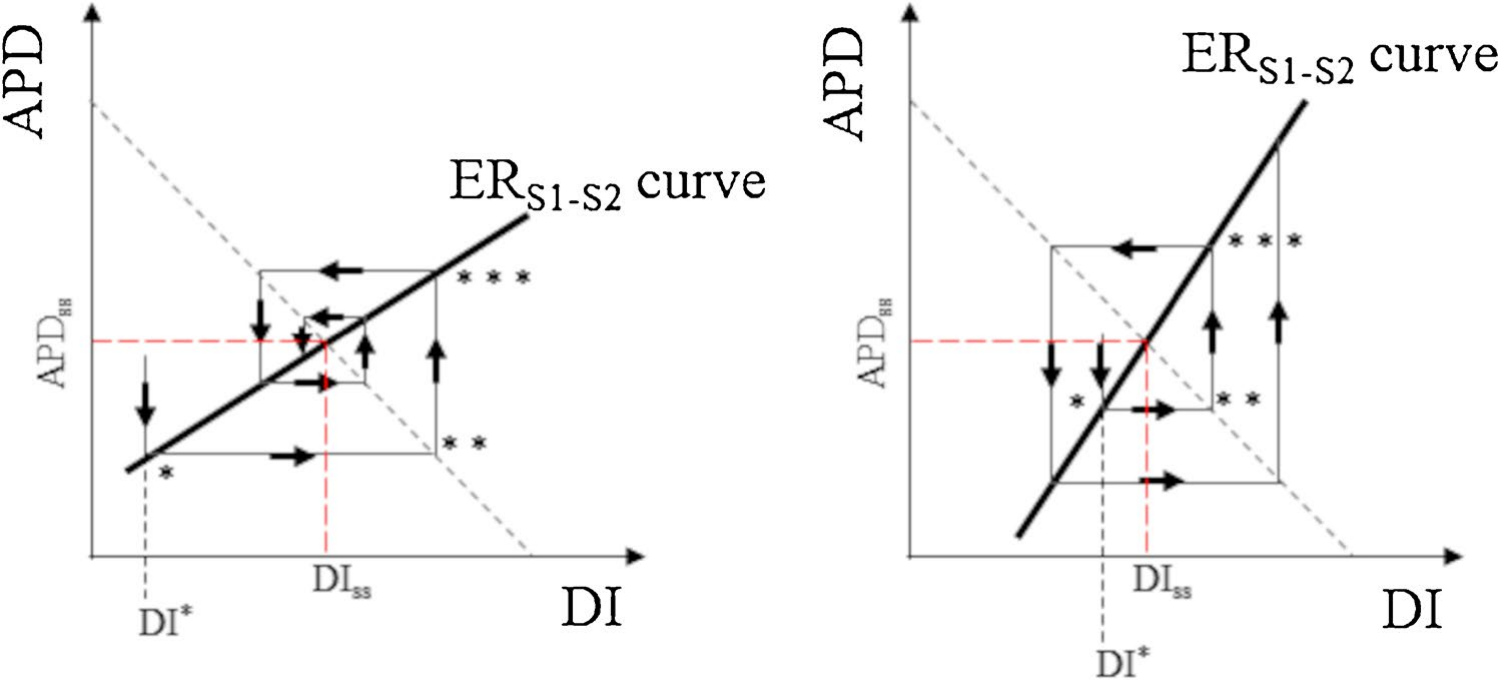
**The restitution hypothesis**. The scheme explains why, during constant BCL pacing, a single perturbation of the pacing rate (DI^*^) can make pacing parameters to alternate, converging or diverging from steady-state values (DI_ss_ and APD_ss_) depending whether the slope of the ER_S1-S2,DI_ curve (thick line) is smaller (left) or greater (right) than 1. The broken grey straight line represents APD = BCL – DI (modified from [65])

The ER_S1-S2_ slope is also a quantitative marker of repolarization stability, as it has been described in a study on microelectrodes impaled frog ventricular strips by Nolasco and Dahlen, who first formulated the so-called electrical restitution hypothesis (ERH) [65, 105]. The theory is borrowed from analogy with electrical negative feedback systems and describes the input (pre-test DI) – output (test APD) relationship when a test beat is anticipated or delayed (S2) with respect to the constant BCL (S1), which is resumed after the test beat. Since at the steady-state BCL is equal to APD_ss_ + DI_ss_ (subscript ss for steady state), then the straight line APD(DI) = BCL – DI intersects the ER_S1-S2,DI_ curve in a point whose coordinates are DI_ss_ and APD_ss_ (Fig. 12). When DI_ss_ is shortened (DI*) by a premature stimulation, the test beat will assume, by definition, an APD value predicted by the ER curve (* in the figure) and, since constant BCL is resumed right after the test, the corresponding DI will be given by the APD (DI) straight line (** in figure). At the new DI value, the ER_S1-S2,DI_ curve will provide, in turn, the new APD value (***), and so on. It can be verified graphically that, if the slope of ER_S1-S2,DI_ curve is < 1 (left panel of Fig. 12), the pacing perturbation leads, over succeeding beats, to smaller APD changes, which lead in turn to smaller DI changes, so that the perturbation is soon quenched into the steady-state APD_ss_, DI_ss_ values. The opposite happens when ER_S1-S2,DI_ slope is > 1 (right panel of Fig. 12), with the perturbation being amplified, instead of quenched, leading to short-long APD alternation. The graphical approach of Nolasco and Dahlen has been formalized by Guevara and colleagues into difference equations [54], which has the remarkable advantage of analyzing salient features of APD alternans based on general dynamics assumptions, even in the absence of a detailed knowledge of the underlying ionic processes [47, 50].

### ER slope as a marker of arrhythmogenicity

Since APD alternans is a recognized precursor of ventricular tachy-arrhythmias, including VF [130, 139], the possibility of predicting these events based on measurable dynamic properties has soon attracted the interest of cardiac physiologists. Over the years, the ERH has been confirmed by experiments and simulations, and maneuvers that reduce the slope of ER_S1-S2_ have been considered a suitable way to contrast cardiac propensity to VF [44, 79, 127, 142, 162, 166] and a promising target for antiarrhythmic drug design [49, 165]. The ERH has been applied, as I will show, in a vast number of physiological and pathological cases where it does predict the increased risk of arrhythmias based on the slope of ER_S1-S2_ curve.

### Failures and shortcomings of the hypothesis

Concerns have been risen on the generality and accuracy of the ERH, and its incompleteness, when not incorrectness, has been demonstrated by several theoretical and experimental studies [48]. Stable 1:1 APD behavior has been reported in fact in conditions where ER slope was > 1 [4, 56], and 2:2 behavior with slope < 1 [46]. The major concern is undoubtedly the fact that ERH depends on the way ER is measured [36]. Since the hypothesis is based on the ER_S1-S2_ mechanism, its use should in principle be restricted to cases where restitution was measured according to S1–S2 protocol. In practice, though, ERH has been frequently applied also to ER_dyn_, rising concerns on the comparability of results. ER_S1-S2_ and ER_dyn_ curves tend in fact to exhibit different steepness (Figs. 4, 5, 6, and 8), thus proving different ability in predicting alternans and VF on the base of ERH [109, 110, 114, 151]. In canine endocardial slices and in isolated Purkinje fibers for example, ER_dyn_ shows a slope > 1 whereas the slope of ER_S1-S2_ curve is < 1, and the former has been proposed as a more informative pro-fibrillatory predictor [79, 127]. A relationship has also been proposed between the two representations, where ER_dyn_ is the main determinant of the slope of ER_S1-S2_ and, in the absence of other variables, factors prolonging steady-state APD are expected to steepen the ER curve (see chapter 8). Even considering only ER_S1-S2_ curves, though, cases are documented in failing human hearts, in which S_max_ simply does not correlate with inducibility of tachyarrhythmia [102, 140], or it is unchanged [81, 102], or even significantly decreased [51, 101, 172] in pro-arrhythmic conditions. A further limitation of the ERH is that it is defined on the perturbation of a constant pacing rate, thus disregarding dynamic properties under variable pacing rate, like cardiac memory, significant to AP repolarization (see chapter 7). Another concern has been risen by Franz and refers to the three-phasic nature of the ER curve (Fig. 4). He argues that the shortening of APD by lengthening of DI during the negative sloping tract of the curve allows the subsequent APD to move more quickly from the steep initial phase onto the flat phase. A less steep initial phase, he suggests, would protract the transition toward more fully recovered APD and, with that, perpetuate electrical alternans and promote arrhythmias [43]. Species and spatial heterogeneity of ER properties, together with the protocol dependence of results, furtherly complicate ERH interpretation [122].

## Contribution of ionic currents and concentrations to ER

Already in early ER_S1-S2_ studies it was clear that the behavior of AP repolarization after a pre- or post-mature beat, was determined by the state of the cell membrane at the time the delayed stimulus was delivered, thus by the recovery state of ionic currents and of intracellular ionic concentrations underlying the LCB [18]. Even though intrinsically intermingled, the first mechanism determines mainly the early phase of the ER curve, whereas the second the later phase at longer CIs [37]. Briefly, as CI (and corresponding DI) shortens, the recovery of the gating state of ionic currents underlying the LCB is partially prevented, particularly that of the inactivation of inward calcium current and of the deactivation of outward potassium current, thus leading to less calcium current and more potassium current flowing during the test beat and, with that, to APD shortening [30, 45, 60, 72].

All the above mechanisms are also involved in ER_dyn_, where intra- and extra-cellular accumulation of ions over several beats also play their role. As DI shortens, for example, less time is allowed for removal of intracellular calcium, which tends to accumulate, decreasing calcium current via calcium dependent inactivation and via decrease of electrochemical calcium gradient, and exerting therefore a shortening effect on APD [17]. Finally, increase in pacing rate leads to extracellular potassium accumulation, particularly within the T tubules, which plays a major role in both ER_S1-S2_ and ER_dyn_ of the cardiac AP [128].

The contribution of specific ionic currents and concentrations to ER is summarized here.

### Ionic currents

The role of ventricular AP plateau currents in determining the form of steady-state ER_dyn_ curve is extensively covered in literature [30, 45, 60, 68, 72, 159, 119, 189]. For a detailed description, it is recommended to refer to comprehensive reviews [17, 125]. Here I will focus on standard ER_S1-S2_ studies and refer to dynamic protocols only when necessary for the discussion.

#### l-type calcium current

**Fig. 13:**
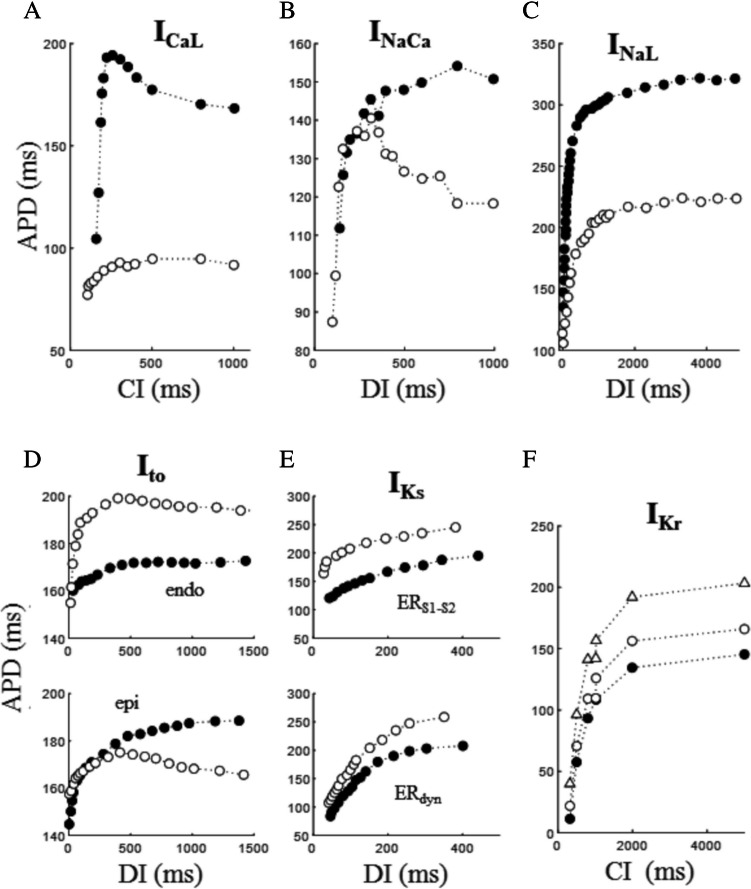
**Role of ionic currents**. In all panels, control conditions are reported as filled dots. **A** Effect on ER_S1-S2,CI_ (BCL = 1000 ms) of blocking I_CaL_ in calcium buffered ferret ventricular cells (data from [68]). **B** Buffering intracellular calcium with BAPTA highlights the role of I_NaCa_ on ER_S1-S2,DI_ recorded in ferret ventricular cells (data extracted from [68]). **C** Effect of blocking I_NaL_ with TTX on ER_S1-S2,DI_ (BCL = 500 ms) in dog Purkinje fibers (data from [37]. **D** The block of I_to_ with 4-AP induced opposite effects in ER_S1-S2,DI_ (BCL = 2000 ms) curves of endocardial and epicardial dog ventricular myocytes (data from [91]. **E** Effect of reducing I_Ks_ with chromanolol on ER_S1-S2,DI_ (BCL = 300 ms) and ER_dyn,DI_ in pig right ventricular tissue (data from [69]. **F** Effect of 40% (empty dots) and 80% (empty triangles) block of I_Kr_ on ER_dyn,CI_ in the Luo and Rudy ventricular model (data from [160]

The role played by l-type calcium current and by its intracellular calcium modulation in ventricular ER_S1-S2_ properties has been investigated since early studies [62, 152]. The recovery of I_CaL_ from inactivation is a key determinant of ER curve in dog [137] and in humans [6]. Janvier and colleagues investigated the involvement of this current in shaping the ER_S1-S2_ curve in rat and ferret microelectrode impaled ventricular myocytes by blocking the current with 20 µM nifedipine (Fig. 13A). Beside the expected AP shortening, the block of I_CaL_ also induced a marked flattening of the ER_S1-S2_ curve [68]. The restitution of the l-type calcium current has also been studied in voltage clamped isolated dog and guinea pig ventricular myocytes by Tseng, who found a major involvement of the sarcoplasmic calcium release in this process [152].

#### Sodium-calcium exchanger current

The reversible electrogenic contribution of NCX to ER is intrinsically intermingled with that of I_CaL_ and of the rest of calcium cycling. The fact that I_CaL_ is the main source of cellular calcium inflow and, in turn, of SR calcium content and that the rise in intracellular calcium tends to turn off the depolarizing source via calcium-dependent inactivation and turn on the depolarizing NCX current, make the two mechanisms extremely difficult to be isolated. Also, at short CIs, calcium transient is depressed [168], leading to decreased depolarizing NCX plateau current and to APD shortening [68], and intracellular sodium accumulates, increasing repolarizing NCX current and contributing to APD shortening as well [160]. The kinetics of I_CaL_ and that of potassium delayed rectifier (~ 30–90 ms) seems to be too fast to account for the early ER phase, whereas that of I_NaCa_ under decrease of calcium transient has a more similar time course (~ 200 ms). In the experiment reported in Fig. 13A intracellular calcium was buffered to separate the effect of I_NaCa_ from that of I_CaL._ Janvier and colleagues have indeed measured I_NaCa_ restitution in microelectrode impaled rat and ferret ventricular myocytes and found that it declines, as CI decreases, with a time course similar to that of ER_S1-S2,CI_ curve [68]. They verified the relevance of this effect by measuring the curve after buffering intracellular calcium with BAPTA, which led to reduction of APD and to a great alteration of the entire ER_S1-S2,CI_ curve (Fig. 13B).

#### Sodium current (late component)

A potent inhibitor of I_NaL_ (GS967) has been shown by Pezhouman and coworkers to shorten APD and flatten ER_dyn_ curve and has been proposed by the same authors as a useful antiarrhythmic drug [121]. Analogous APD shortening and flattening of ER_dyn_ curve have been obtained by Morita and colleagues by blocking I_NaL_ with ranolazine in Langendorff perfused rat hearts [99] thereby converting sustained to non-sustained VF. The same effect, though measured on ER_S1-S2_, has been shown by Elharrar in dog Purkinje fibers [37] (Fig. 13C).

#### Transient outward potassium current

Litovsky and Antzelevitch studied the difference in APD response to 4-AP between epicardium and endocardium in microelectrode impaled dog right ventricular myocytes. AP waveforms of the two cell types rely differently on I_TO_, whose block produces opposite modifications in their ER_S1-S2_ curves (Fig. 13D), which is likely to affect intramural reentry and rate dependency of ventricular arrhythmias [91]. The different effects on ER_S1-S2_ curve of blocking I_TO_ in ferret normal and calcium buffered ventricular myocytes have also been studied by Janvier and colleagues [68].

#### Delayed rectifier potassium current

The differential role of the two components of the delayed rectifier potassium current, I_Kr_ and I_Ks_, on ER_dyn_ has been studied in the Luo and Rudy numerical model [92] of the cardiac ventricular AP [160, 189]. Zeng and colleagues found that, at shorter DIs, the two currents are the major determinants of APD restitution, with I_CaL_ only playing a minor role, whereas longer DIs are dominated by long lasting changes in intracellular calcium and, in turn, by calcium dependent inactivation of I_CaL_ [189]. The role of I_Ks_ in both ER_dyn_ and ER_S1-S2_ properties has been studied by Jing and colleagues in pig right ventricular tissue by respectively reducing (chromanolol) or increasing (mefenamic acid) this current [69]. The reduction effect is reported in Fig. 13E. The reverse rate-dependent effect of I_Kr_ on ER_dyn_ (Fig. 13F) has been attributed by Wu and colleagues to the simultaneous decrease of I_NaL_ for smaller CI values, which is significant to bradycardia-related ventricular arrhythmias [175].

### Ionic concentrations

#### Calcium

**Fig. 14:**
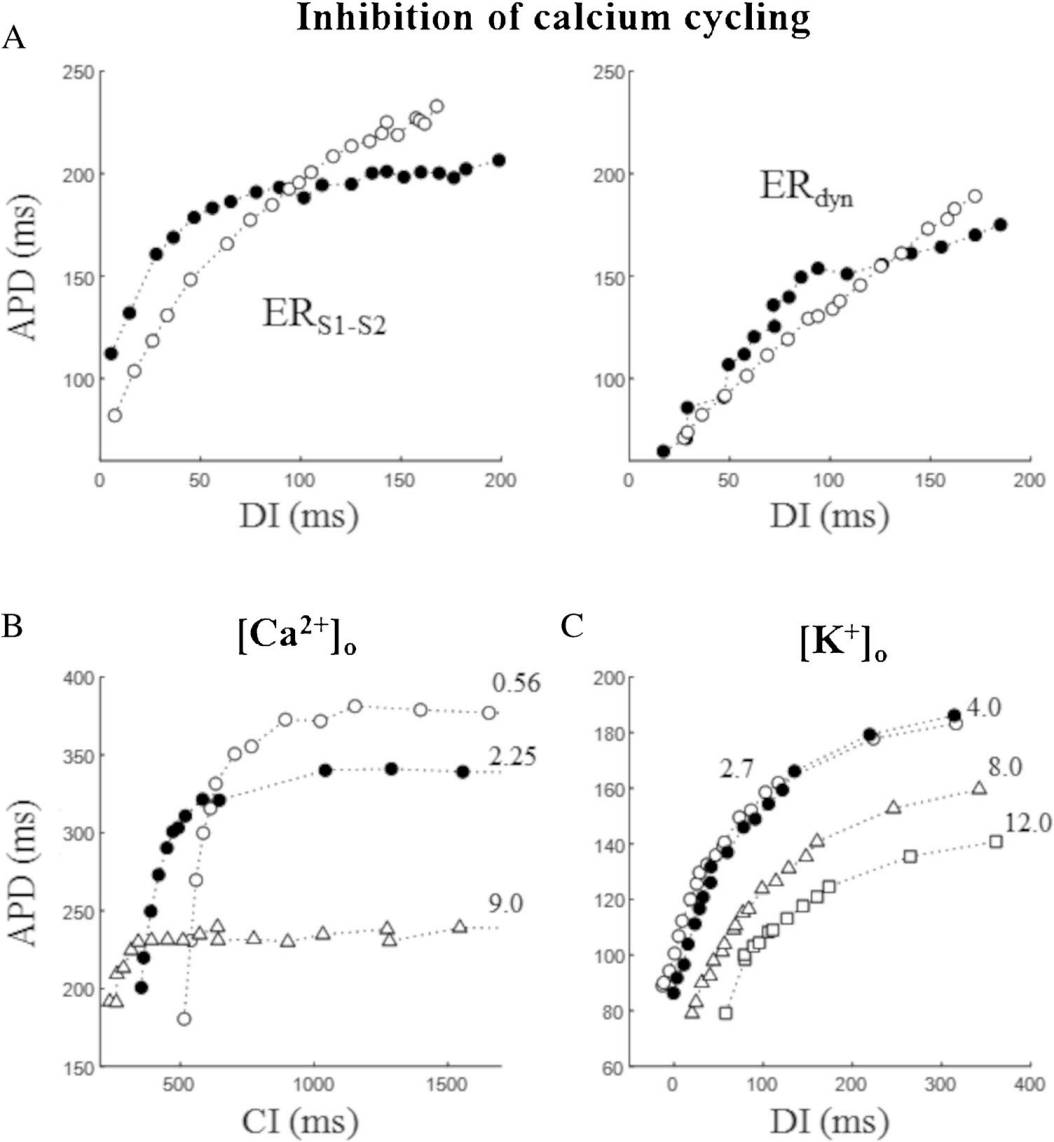
**Role of ionic concentrations**. In all panels, control conditions are reported as filled dots. **A** Effect of thapsigargin + ryanodine on ER_S1-S2_ and ER_dyn_ in patch clamped rabbit myocytes (data from [52]. **B** Effect of varying extracellular calcium concentration (mM) on ER_S1-S2,CI_ recorded in cat papillary muscle (data from [17]. **C** Effect of varying extracellular potassium (mM) on ER_dyn,DI_ measured in dog ventricular tissue (data from [80]

The role of intracellular calcium cycling in ER has been investigated in perforated-patch clamped rabbit ventricular myocytes by Goldhaber and colleagues. They studied the effect of suppressing intracellular calcium transient by simultaneously inhibiting SR calcium re-uptake and release, respectively, with thapsigargin and ryanodine. They found that calcium cycling inhibition flattens ER_dyn,DI_ curve but not ER_S1-S2,DI_ (Fig. 14A) and proposed that calcium cycling is the major responsible for the differences in the two cases, via a memory effect (see chapter 7) due to pacing-dependent calcium accumulation [52]. Also, Szigligeti and colleagues have found a dramatic difference in intracellular calcium sensitivity of ER_S1-S2_ curve in microelectrode impaled ventricular tissue of rabbits, where elevation and reduction of intracellular calcium concentration shifted, respectively, downward and upward, the ER_S1-S2,DI_ curve [143]. As explained above, reduction of DI for shorter CIs shortens APD via incomplete removal of voltage-dependent inactivation of I_CaL_. At shorter DIs though, unremoved intracellular calcium also causes reduction of I_CaL_ via the reduction of the electrochemical calcium gradient and via increased calcium-dependent inactivation [126], thus contributing to APD shortening. On the other hand, the recovery of calcium-dependent I_CaL_ inactivation is quite rapid at the diastolic potential and cannot, by itself, account for the entire APD shortening for shorter CIs, where I_NaCa_ significantly contributes.

Calcium concentration affects ER dynamics also extracellularly, particularly early ER phase, as shown by Bass in his study on microelectrode impaled cat papillary muscle [8, 9]. Very interesting is the observation made by Boyett and Jewell on calcium modulation [17] in their work on cat papillary muscle. When they measured the effect of different extracellular calcium concentrations, which are known to tightly control APD, they found that, indeed, APD shortened with increasing extracellular calcium, thus shifting downward the corresponding ER_S1-S2,DI_ curves but, importantly, leaving their time constants unmodified (Fig. 14B).

#### Potassium

The increase in pacing rate leads to potassium accumulation in restricted extracellular spaces, which, via depolarization of the cell membrane and shortening of APD, is a major responsible of potassium dependence of ER [76]. However, Robinson and colleagues have challenged this view by showing that potassium dependence of ER properties hold also when measured in isolated ventricular cells, lacking therefore intercellular cleft, or in isolated Purkinje cells, lacking T tubules, and cannot therefore rely, at least not completely, on restricted extracellular potassium accumulation [128]. The effect of extracellular potassium concentration on ER_S1-S2,DI_ curve has been measured by Koller and colleagues on microelectrode impaled dog ventricular tissue [81]. They found that the increase of extracellular potassium from 2.7 up to 12 mM decreased the slope of ER_S1-S2,DI_ curve at long but not at short DIs (Fig. 14C), which might explain why hyperkalemia suppresses ventricular fibrillation.

A combination of ionic mechanisms also explains the three-phasic behavior of ER_S1-S2_ curve (Fig. 4) in the ventricular tissue of several mammalians [14, 41, 97, 98]. Franz has proposed a three ionic currents model where the earliest ER phase, up to the relative maximum, is mostly determined by the recovery of fast sodium current, the subsequent declining phase by insufficient activation of l-type calcium current, and the final monotonic rising phase due to the contribution of rapid and slow components of outward potassium current [43]. In a theoretical work, Bernus and colleagues [14] studied separately the arrhythmogenic role of the early positive and the negative limb of the curve and found that increasing the slope of the descending part or the amplitude of the bi-phasic part increases the meandering of originating spiral waves, with significance to formation of complex arrhythmias and cardiac fibrillation.

## AP memory and restitution

### Long-term memory and the restitution portrait

Long-term cardiac memory (LTM) refers to long-lasting (minutes–hours) effects on both AP depolarization and repolarization operated by altered and prolonged pacing conditions [129, 141], and is measured at the body surface level as modification of the electrocardiographic T wave that persists after resumption of normal pacing. It has been shown that AP repolarization stability depends on LTM [70], and that the incorporation of this memory effect into numerical predictive models [40, 116, 148, 164] allows to explain why ERH fails in many experimental and clinical instances. Whereas ER_S1-S2_ measures the instantaneous response to changes in BCL and ER_dyn_ the steady-state response, the constant BCL protocol ER_CB_ describes the transition between the two, and the role of ER_CB_ slope in determining the stability of AP repolarization has been shown by theoretical models [116, 148]. Ideally, to capture the entire APD dynamics when constant pacing rate is perturbed, including instantaneous and LTM effects, one would need to measure all three ER_S1-S2_, ER_dyn_, and ER_CB_ in the same preparation. This is what Tolkacheva and colleagues have proposed by means of the so-called pacing-perturbed down-sweep protocol [70, 148], whose results, as described above, can be compactly summarized into the restitution portrait.

### Short-term memory and beat-to-beat restitution

ER_S1-S2_, ER_dyn_, and ER_CB_ (and therefore ER portrait) refer to the dynamic properties of an excitable membrane under constant pacing conditions. When pacing rate is beat-to-beat variable, for example when CL is made varying randomly [22, 29, 34, 81, 90, 180, 183–185], or following a sinusoidal law [1, 55, 178, 123, 183–185], or alternating between two values [13], or when it changes linearly, for example, following a saw-tooth waveform [180, 184], restitution dynamics can be studied through the ER_btb_ curves (Fig. 11). The same representation has been used in spontaneously beating hearts, either measured as APD versus preceding CL, or as electrocardiographic QT versus preceding RR, and proposed as a viable biomarker of arrhythmia vulnerability [39]. I have characterized and compared ER_btb_ properties in rat ventricular myocytes, and in several human ventricular numerical AP models in connection with short-term memory (STM) and repolarization stability [180, 183–185].

STM refers to the dependence of cardiac AP from a given number of preceding excitation markers, like APDs, DIs, or even intracellular ionic concentrations, i.e., to the ability of an AP to remember its recent pacing history [1, 35, 149]. Remembering, on the other hand, means storing information somewhere and, in the case of STM, we can think the information stored into the activation and inactivation kinetics of ionic channels and into their time, voltage, or intracellular ion concentration dependence. Unlike long-term cardiac memory, STM is removed as soon as the beating stops for few beats or, more physiologically, when it reaches a steady state, and can in fact be estimated by measuring the time taken for APD to reach steady-state value after an abrupt change of constant pacing CL [94, 185, 188]. STM, in other words, measures the difference between a sudden and a steady-state response to a pacing CL change between two different constant values and, as such, is not captured by ER_S1-S2,DI_ nor by ER_dyn,DI_ curves [5], but it can be measured from the angle between the two [42, 94].

A link has been demonstrated between STM and the stability of ventricular AP repolarization, as the increase in the former frequently increases the latter [20, 55, 173, 183–185], and the initiation of ventricular arrhythmias has been reported to be affected by both ER properties and STM [20]. In the case of CL harmonically oscillating within a given range *σ* around a constant BCL, and with an angular frequency *ω* [183–186], the linking mechanism between STM and stability has been related to the hysteretic behavior that ER_btb_ curves assume under these pacing conditions for large *ω* values [1, 12, 173, 183–186], and which is functional to quench APD oscillations and potentially prevent malignant arrhythmias [12, 183]. Under these pacing conditions, in fact, the ER_btb_ goes from a simple monotonic curve for pacing rate constant or oscillating at low ω values, to a hysteretic loop for higher ω values (Fig. 11A–B, third column), where the vertical width of the loop (W_CL_ in figure) measures STM, increases under block of I_Kr_ [1] or I_Ks_ [183], and decreases under I_CaL_ block [55, 183]. Under non-steady-state fast pacing conditions, instead of moving on a single ER curve, subsequent beats (their coordinates APD,CL) keep moving on a family of ER curves, whose vertical displacement determines W_CL_ (Fig. 11A–C, third column) and, with that, the amount of STM and APD stability [35, 184, 185]. Mechanistic models should incorporate STM as well to explain discrepancies between stationary and dynamic ER properties [38].

At the whole organ level, the reduction of heart rate variability (HRV) frequently indicates pathological conditions and is a good predictor of mortality following acute myocardial infarction [146]. Dvir and Zlochiver have shown that the correlation between HRV and repolarization stability is associated with STM accumulating during beat-to-beat variable pacing rate [33–35], which is revealed at the cellular level by the simulated experiments reported in Fig. 11D, where APD alternans at constant pacing rate was removed by switching to fast beat-to-beat oscillating, either sinusoidally (left panel) or randomly (right panel), CLs.

## APD dependence of ER

### A largely verified hypothesis

**Fig. 15:**
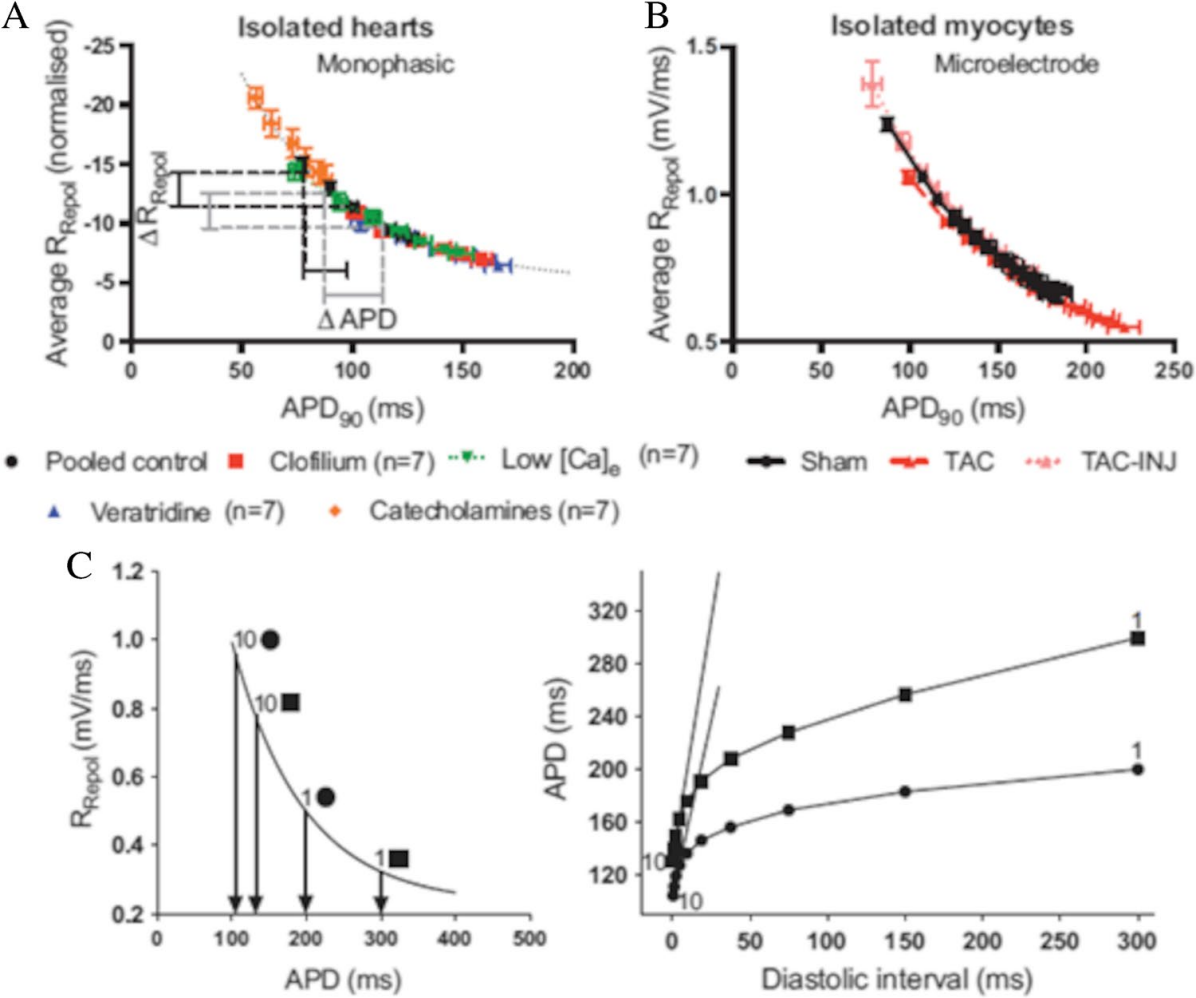
**APD dependence of ER**. **A** MAP experimental data showing that APD is non-linearly related to the average rate of repolarization in isolated guinea pig hearts, either in control conditions or under different APD altering conditions (see color codes). **B** Same dependence measured in microelectrode recorded APs from guinea pig isolated ventricular myocytes. The relationship holds in cells from aortic constricted hearts (TAC), and in the same cells injected with constant current(TAC-INJ). ER_dyn,DI_ curve (**C**, right) was simulated by assuming DI shortening as step increases in R_repol_ from 2 different starting APDs (denoted by 1) and reporting the corresponding APDs taken from the R_repol_ – APD relationship (**C**, left) (figure from [134]

In a recent work, Shattock and colleagues have shown that the duration of the cardiac AP, either measured in Langendorff-perfused hearts or in enzymatically isolated myocytes, depends non-linearly on the repolarization rate [171] (Fig. 15A and B), i.e., on the total amount of ionic current flowing across the membrane, and how this relationship explains why AP prolongation promotes electrical instability. This way to look at longer APs as determined by less total repolarizing current would unify, according to these authors, the interpretation of several controversial phenomena, including the reverse rate dependence (RRD), i.e., the fact that APD prolonging agents are more effective at slow, rather than high, pacing rate. In a further study on isolated hearts and ventricular myocytes from guinea pigs and rabbits, they describe the relevance of this interpretation to ER [134]. The observation that the steepness of the ER_dyn_ curve tends to correlate with the value APD assumes at the longest DIs [36, 69, 91, 109, 110, 145, 164, 177] is a mere consequence, according to Shattock, of its non-linear dependence from the maximum rate of repolarization (Fig. 15). They verify this hypothesis under a plethora of conditions, including normal, pathological, and pharmacological states, to the point that it has been proposed as a new fundamental law in cardiac electrophysiology [26]. If steady-state APD is the main determinant of the slope of ventricular ER_dyn_ curve, any factor that prolongs it would be expected to steepen the curve, which explains the failure of ERH in several experimental and clinical instances, including the effect of class III antiarrhythmic drugs on restitution (see below).

### Concerns with the hypothesis

The dependence of ER from APD does explain many experimental observations but it suffers, in my opinion, from two basic theoretical shortcomings. First, it is described for ER_dyn_ but then equally referred to ER_dyn_ and ER_S1-S2_, which, as I have shown, are different in their features (Figs. 4, 5, 6) and in their ability to predict repolarization stability. Second, as Shattock and colleagues note, their hypothesis raises the very fundamental question, on whether the dynamics of ER and steady-state APD can be dissociated, and they conclude they cannot, being the former a necessary consequence of the latter. I have investigated this issue in two theoretical studies in which I have shown that, indeed, the two can be dissociated. I have found that two (or more) different sets of parameters, like maximum ionic channels conductances or half maximum activation potentials, of the equation system describing a specific ventricular AP can be obtained within a physiological range, and generate identical AP waveforms, though endowed with quite different properties [181]. In particular, they show different electrical and pharmacological modulation and, notably, different restitution properties [181, 182], which contradicts Shattock’s view of a biunique relationship between APD and ER slope. I have demonstrated the extent of this effect in guinea pig and human ventricular AP models, but the phenomenon is altogether general, as a given AP waveform does not guarantee the existence of a corresponding unique set of equation parameters [32]. Thus, although APD is frequently a good predictor of ER steepness, this is not true in general, which might be relevant in interpreting apparently contradictory and paradoxical cases, including the effect on ER of spatial and temporal variability of APD [25, 179].

## Physiological and pathological modulation of ER

Any physiological, pathological, or pharmacological state involving changes in the ionic mechanism underlying ventricular EC coupling will modify AP trajectory and/or its dynamics, thus potentially affecting ER properties. Consequently, restitution has been studied in several conditions, including LQT syndrome [177], Brugada syndrome [61], diabetes [153], just to name a few. I will limit my description to the cases, listed below, in which ER properties have provided more useful descriptive and predictive knowledge.

### Autonomic modulation of ER properties

**Fig. 16:**
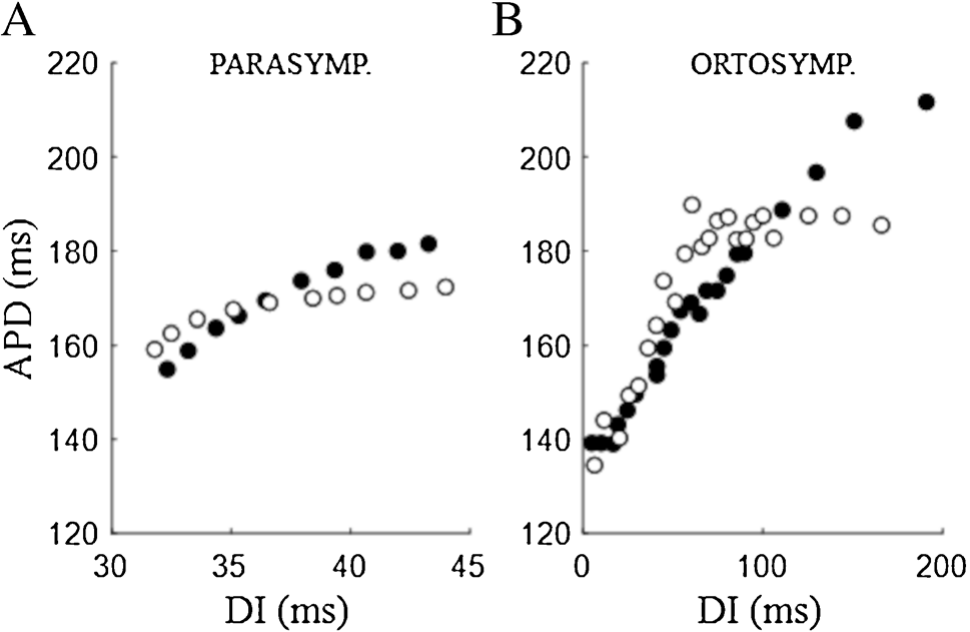
**Autonomic modulation**. Control conditions are reported as filled dots in both panels. **A** Effect of the stimulation of the auricular branch of the vagus nerve in epicardially MAP-recorded ER_S1-S2,DI_ curve (BCL = 350 ms) of infarcted dog. S_max_ of the reported curves goes from 1.78 in control to 1.27 under vagal stimulation (data from [104]). **B** Effect of infusion of isoprenaline in MAP-recorded ER_S1-S2,DI_ curve (BCL = 400 ms) of the right ventricular septum of a patient. S_max_ of the reported curves goes from 0.707 in control to 1.120 during isoprenaline infusion (data from [145]

ERH holds well in explaining autonomic modulation of arrhythmia inducibility. Its role in the antiarrhythmic action of vagus nerve stimulation has been shown by Nasi-Er and colleagues, who found that the stimulation of the auricular branch of the nerve in infarcted dogs decreased the S_max_ of ER_S1-S2,DI_ (Fig. 16A) and, with that, enhanced the threshold for VF [104]. Conversely, the pro-arrhythmic action of adrenergic stimulation has been studied by Taggart and colleagues who showed that isoprenaline and adrenaline increase the steepness of ER_S1-S2,DI_ curve measured from MAP recordings in the right ventricle of patients [145] (Fig. 16B). Also, renal sympathetic denervation, by reducing systemic sympathetic activity, has been found to reduce ER_S1-S2,DI_ slope and the occurrence of arrhythmias during acute myocardial ischemia [64].

### Ischemia and hyper-kalemia

**Fig. 17:**
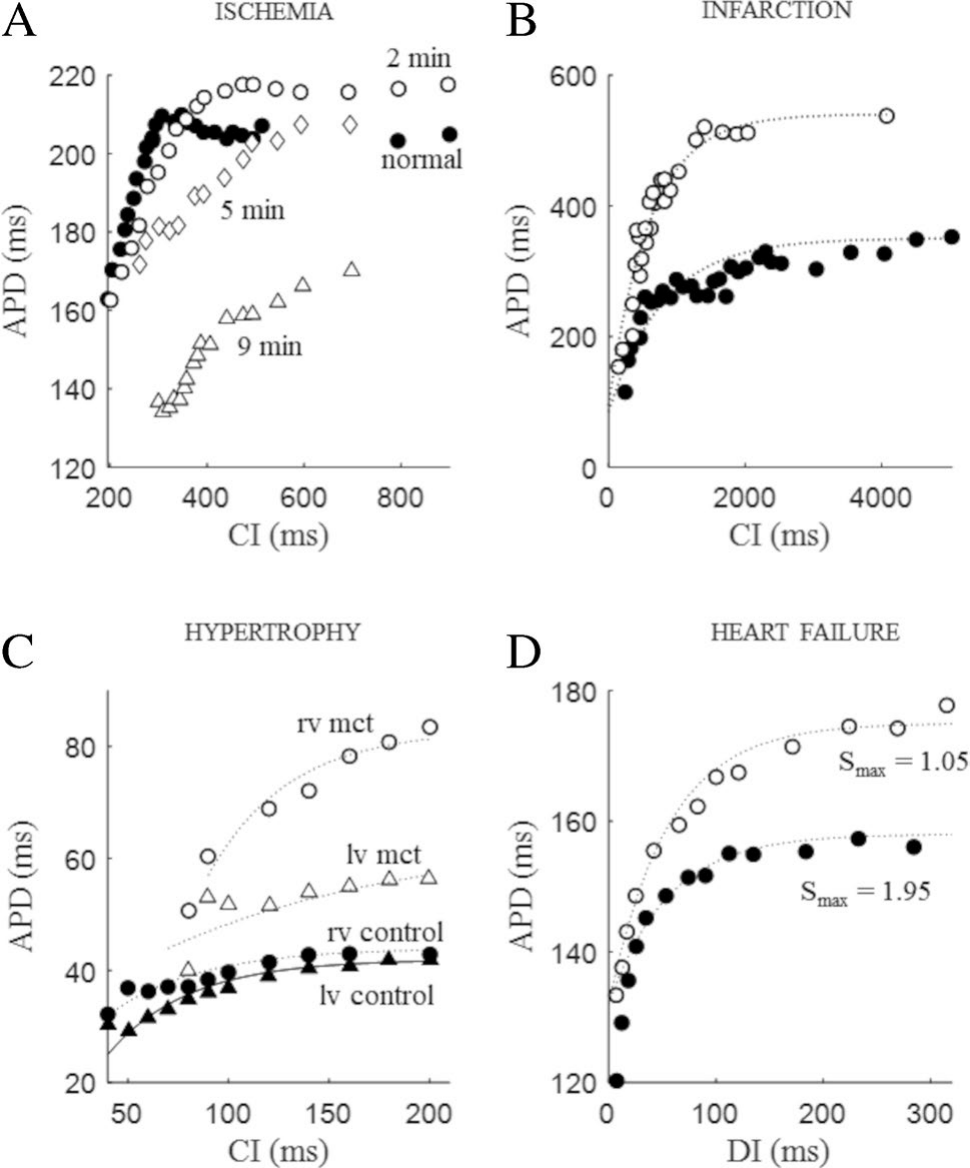
**Pathological states**. **A** ER_S1-S2,CI_ curves (BCL = 500 ms) measured via epicardial MAP recordings at a single ventricular site in a Langendorff-perfused rabbit heart in control condition (filled dots) and at progressively increasing times (values reported in figure) after induction of global ischemia (data from [86]. **B** ER_S1-S2,CI_ curves (BCL = 500 ms) measured via microelectrode recordings in single isolated dog Purkinje cells from normal (filled dots) and infarcted hearts (data from [15]. **C** ER_S1-S2,CI_ curves (BCL = 200 ms) via MAP recordings from right (circles) and left (triangles) ventricles in normal (filled symbols) and hypertrophied (empty symbols) rat Langendorff-perfused hearts. Hypertrophy was induced by monocrotaline (mct) injection (data from [11]. (D) ER_S1-S2,DI_ curves (BCL = 300 ms) via epicardial MAP recordings from Langendorff-perfused control (filled dots) and failing (isoprenaline-induced heart failure) guinea pig hearts (data from [140]

Potassium accumulation in restricted extracellular spaces has long been recognized for its contribution to the rate dependence of ventricular APD [76], and local hyper-kalemia following acute ischemia among the major responsible in promoting VF [136, 167, 176]. Notably, hyper-kalemia, beyond depolarizing membrane potential, slowing conduction, and altering refractoriness, has been shown to reduce ER_dyn,CI_ slope [96]. A reduction of ER_dyn,DI_ slope has been found by Koller and colleagues in microelectrode impaled dog ventricular fibers, by incrementally increasing extracellular potassium [80]. The flattening effect of ischemia on the ventricular ER_S1-S2,DI_ curve has also been demonstrated by Taggart on patients by means of MAP recordings from the right ventricular septum during balloon occlusion of the left descending coronary artery [144]. Ischemic-induced reduction of ER_S1-S2,CI_ slope like that in Fig. 17A, according to the ERH, should increase repolarization stability, and therefore contradicts the increased propensity of the ischemic myocardium to develop APD alternans [43, 86]. Several hypotheses have been explored to explain this contradiction.

ER_S1-S2,CI_ curves from MAP recordings have been measured by Dilly and Lab in exposed hearts of anaesthetized pigs, in and around ventricular areas where ischemia was induced by coronary constriction [31]. Following ischemia induction, ER_S1-S2,CI_ curve showed an initial depression in plateau and reduction in magnitude, an transient inversion of these changes, followed by further reduction. The authors propose that development of alternans and VF was due, in this case, to the interplay between ER properties and changes in intracellular calcium dynamics.

Sidorov has also shown that not only hyper-kalemia per se, but the spatial heterogeneity of extracellular potassium promotes repolarization instability in a Langendorff-perfused rabbit heart model, where he created local potassium gradients and optically recorded underlying electrical activity [136]. Finally, Fenton and colleagues have shown in a theoretical study how STM dependence of ER explains the pro-arrhythmic properties of ischemia-induced flattening of the ER_S1-S2,DI_ curve [38]. Restitution portraits were also used to estimate STM under acute myocardial ischemia in a guinea pig ventricular AP model [94]. They showed that ischemia induced a significant decrease of STM, mainly due to the decrease in extracellular calcium rather than to changes in membrane currents. To note, ischemia-induced APD alternans was prevented in coronary-constricted dog hearts by renal sympathetic denervation, which prolonged APD and reduced ER_dyn,DI_ slope [64].

### Hypo-kalemia

The decrease in extracellular potassium also affects the dynamics of ventricular repolarization and, particularly, the ER properties. In the guinea pig heart, for example, hypo-kalemia was found to increase endocardial spatial gradients of ER_dyn,DI_ properties, whereas the same gradients were unaffected in dog and mouse [151]. Hypo-kalemic conditions (3.0 mM) have also been shown to induce steepening of ER_dyn,DI_ curve, thus increasing propensity to develop APD alternans and arrhythmias in Langendorff-perfused murine hearts [132].

### Infarction

As opposed to ischemia, myocardial infarction leads to more consistent effects on the ER curve, by causing an overall increase in its slope, as shown in the ER_S1-S2,CI_ data measured by Boyden in infarcted rabbits (Fig. 17B) [15]. By mapping MAPs at the border zone of infarcted area in the ventricles of open-chest anesthetized dogs, Ohara and colleagues have also shown that, in this region, the S_max_ of ER_dyn,DI_ was significantly higher, and the DI interval over which it was > 1 much larger, which can explain, according to the ERH, the higher incidence of wavebreak [106]. Similarly, Chou and colleagues have hypothesized that subacute myocardial infarction, induced in rabbit hearts by coronary artery ligation, causes electrical remodeling that alters APD restitution and calcium dynamics in the peri-infarct zone [23] where in fact, they measured steeper ER_S1-S2,DI_ curves and unstable calcium dynamics via optical mapping. Moreover, Krog-Madsen and Christini have shown in a simulation study that, when ventricular tissue is made heterogeneous by the presence of a structural barrier like in the case of myocardial infarction, discordant alternans occur during fast pacing due to the heterogeneity of ER_dyn_ properties [83]. Finally, Decker and Rudy have shown abnormal ER_S1-S2_ properties in a one-dimensional numerical model including normal and post-infarction electrically remodeled APs [28].

### Hypertrophy

Though earlier studies have found left ventricular hypertrophy associated with prolongation or no changes in the time constant of ER_S1-S2,DI_ [24, 27], which is consistent with the quicker l-type calcium current restitution found in the same model [131]. Similarly, Benoist and colleagues [11] have found an increase in the ER_S1-S2,CI_ slope, measured with MAPs in Langendorff-perfused rat hearts, where right ventricular hypertrophy was induced (Fig. 17C).

### Heart failure

Increased susceptibility to arrhythmias in heart failure is frequently due to repolarization abnormalities, which include prolongation of ventricular APD and impaired rate dependence [67]. In a guinea pig model of heart failure, induced by sustained adrenergic activation, Soltysinska and colleagues have measured electrical remodeling by recording MAPs in Langendorff-perfused hearts [140]. They found prolongation of left ventricular epicardial AP and an upward shift of the ER_S1-S2,DI_ curve over a wide range of DIs (Fig. 17D). In contrast with ERH, and similarly to other studies contradicting the hypothesis [81, 102], Nanasy et al. 1996, [51, 172], they also found a significant reduction of the S_max_ of the ER_S1-S2,DI_ curve in the failing hearts (from 1.95 to 1.05). A reduction of S_max_ was also found, though in the ER_dyn,DI_ curve, in wedges of ventricular wall from human failing hearts, where APDs were measured by optical mapping [51]. Also, whereas ER_dyn,DI_ slopes were found heterogeneously distributed in the non-failing free wall [51], with a steeper slope from the mid-myocardial area, compared to the sub-epicardium and sub-endocardium, failing hearts showed equal slopes in the three regions. To note, the average slope throughout the mapped area was > 1 for the non-failing, and < 1 for the failing hearts, which emphasizes once again the incompleteness of the ERH as unique criterion of propensity to arrhythmias.

Qualitatively different results, though in a quite different experimental setting, were found by Watanabe and colleagues in a dog model of cardiac failure. In exposed hearts of ventilated dogs, they measured activation-recovery intervals (ARI) from unipolar electrograms recorded from the entire epicardial surface [163] and found that the slope of ER_dyn,DI_ of ARI was significantly steeper in the failing hearts, particularly in the apex and at short DIs. Even though the augmented slope in the failing hearts was less than one, reaching a value of 0.74 at the apex, ventricular extra-stimuli induced VF, whereas no fibrillation was induced by extra-stimuli in non-failing hearts. In a numerical mono-domain model of heart failure, Ponnaluri and colleagues [82, 122] reproduced the increased susceptibility to arrhythmias of the failing heart under rapid pacing which, on the other hand, was not paralleled by steepening of ER_dyn,DI_. Hardy and colleagues have investigated the mechanisms underlying steepened restitution in a rat model of right ventricular failure [58] where they found that the increase in susceptibility to arrhythmias was due to the mechanical restitution changes introduced by reduced l-type calcium current and increased calcium extrusion by NCX.

## Antiarrhythmic drugs

The onset and maintenance of re-entrant arrhythmias depends largely on the cardiac wavelength, the product of conduction velocity and refractory period, and factors decreasing the first or shortening the second facilitate arrhythmia development. An ideal antiarrhythmic drug will prolong APD and do so to a greater degree at fast pacing rate, such as during ventricular tachyarrhythmia [171]. On the other hand, most antiarrhythmic drugs exert their effect in a RRD manner [87, 155, 187] and become less effective during tachyarrhythmias [26]. Studying the effects of antiarrhythmic drugs on ER is complicated by the differences in intrinsic pacing rate and AP dynamics in the different animal models, by the different information from the different restitution protocols (Arpadffy-Lovas et al. 2020), particularly ER_S1-S2_ and ER_dyn_ [114], and by the differences between chronic and acute exposure. Despite all shortcomings and paradoxical results, the effect of antiarrhythmics on the ER properties is still a recognized marker in determining the safety profile of these drugs and will be discussed here for antiarrhythmics of class I-IV of the Vaughan-Williams classification.

### Class I

By interfering with the cardiac sodium current, drugs of this class modify both initial phase and waveform of the ventricular AP and have long been studied for their effects on ventricular ER. Though, as sodium current blockers, they are primarily expected to shorten APD, they sometime prolong it via a secondary inhibition of I_Kr_ [66].

**Fig. 18:**
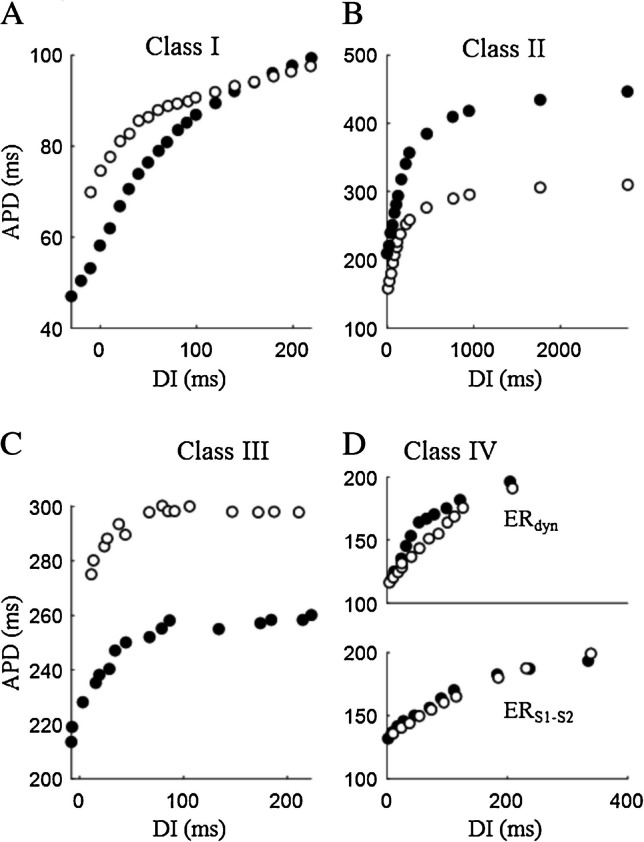
**Antiarrhythmic drugs**. In all panels, control conditions are reported as filled dots. **A** Effect of flecainide superfusion on the ER_S1-S2,DI_ (BCL = 500 ms) measured from microelectrode impaled dog false tendons (data from [154]. **B** Effect of sotalol superfusion on the ER_S1-S2,DI_ (BCL = 500 ms) measured from microelectrode impaled dog Purkinje fibers (data from [88]. **C** Effect of chronic treatment with amiodarone on the ER_S1-S2,DI_ (BCL = 600 ms) measured from MAP recordings in the right ventricle of human patients [115]. **D** Effect of verapamil superfusion on ER_dyn,DI_ and ER_S1-S2,DI_ curves (BCL = 300 ms) measured from microelectrode impaled excised sections of dog ventricle [127]

In their microelectrode studies on excised pieces of dog ventricular tissue and canine Purkinje fibers, Varro and colleagues described the action of several class I antiarrhythmic drugs on the ER_S1-S2,DI_ curve [154, 156, 157, 159], Lathrop & Varro 1990, [100]. All drugs shifted the initial part of the curve toward longer APD values and tent to abolish its early steep part, the larger effect achieved with flecainide (Fig. 18A) and the smallest with lidocaine. Analogous results were found in guinea pig papillary muscle [117] and in Langendorff-perfused dog hearts [59]. The slope decrease induced by flecainide on the early part of ER_S1-S2,CI_ curve has also been measured by Malfatto and coworkers in microelectrode impaled Purkinje fibers, and explained with the faster effect of this drug on the cardiac I_K_ with respect to that on I_Na_, which takes few beats to develop and has less impact on the test AP. Propafenone had no effect on the ER_S1-S2,CI_ curve in the same preparation [93].

The flattening effect of lidocaine shows its antiarrhythmic potentiality in the case of hypo-kalemia. The decrease of extracellular potassium down to 3.0 mM led in fact, in Langendorff-perfused murine hearts, to steepening of the ER_dyn,DI_ curve and increasing propensity to develop APD alternans and arrhythmias, which were prevented by lidocaine [132]. In microelectrode impaled dog cardiac Purkinje fibers, Elharrar has shown that lidocaine and mexiletine prolong the time constant of the fast early component of APD restitution [37]. Also, by shifting upward and flattening the early part of the ER_dyn,DI_ curve, procainamide prevents critical shortening of APD and eliminates tendency to spontaneous wave break underlying maintenance of VF [74]. Osadchii’s work on MAPs recorded in perfused guinea pig hearts confirms the procainamide induced upward shift of ER, which he attributes to I_Kr_ inhibition, but also documents that procainamide, and not lidocaine, increases the maximal value of ER_dyn,DI_ slope, and produces arrhythmogenic effects in the clinical setting. He mainly attributes this finding to the heterogeneously distributed density of this current within the ventricular wall and between right and left ventricles, which determine in turn heterogeneity in ER_dyn_ properties [111]. Also, when measured in canine endocardium with standard microelectrodes, procainamide did not modify ER_S1-S2,DI_ nor ER_dyn,DI_ slope [127]. In his comprehensive study on monophasic APs recorded in perfused guinea pig hearts, Osadchii compares several different class I antiarrhythmics to explain why some of them (flecainide and quinidine) increase the propensity of ventricular tachy-arrhythmias, whereas others (lidocaine and mexiletine) do not [112]. Altered restitution properties were found in right ventricular endocardial tissue of dogs in which a previous treatment with intravenous quinidine induced ventricular tachycardia and VF (Karaguezian et al. 1993). In this case, together with the upward shift of the ER_S1-S2,DI_ curve, hearts showed a steepening of the early part of the curve, to which the authors attribute the increased instability of APD dynamics leading to alternans and fibrillation. Also, ranolazine significantly reduces S_max_ of microelectrode recorded APD ER_dyn_ curve and converts sustained to non-sustained VF in isolated rat hearts [99].

### Class II

Drugs of this class inhibit beta-adrenergic activation of adenylate cyclase and mainly exert their action by slowing pacemaker activity [53], though frequently showing class I and class III properties [120]. Sotalol, for example, has been shown, in dog cardiac Purkinje fibers, to shift upward the ER_S1-S2,DI_ curve, increase its initial slope, without modifying restitution rate [88, 89] (Fig. 18B). A sotalol-induced increase in ER_dyn,DI_ slope was also shown in normal epicardium of dogs, where, despite the pro-arrhythmic increase in slope, the drug prevented AP alternans at short DIs [118]. Propranolol slowed the fast component of APD restitution and shifted the ER_S1-S2,DI_ curve toward longer APDs in dog cardiac Purkinje fibers and ventricular muscle [159].

### Class III

Drugs of this class block one or more of the repolarizing potassium currents leading to prolongation, most often rate dependent, of ventricular APD. Discrepancies are found not only in the effect of different class III drugs on ER properties but also in the effect of the same drug on different preparations.

Amiodarone, for example, has been found to increase APD and curtail ER_S1-S2,DI_ curves in a rate dependent manner, without altering the slope, in pre-treated rabbit hearts [75]. Similarly, chronic administration of amiodarone prolonged monophasic APD recorded in the right ventricle of patients, where, on the other hand, it flattened the ER_S1-S2,DI_ curve [115] (Fig. 18C). In contrast, amiodarone perfusion did not change steady-state APD nor restitution in guinea pig papillary muscle, whereas chronic pretreatment led to APD prolongation and to decrease of the initial slope of ER_S1-S2,DI_ curve [157]. Similarly, shortening of the ventricular APD and slowing of the ER_S1-S2,DI_ were measured in microelectrode impaled dog Purkinje fibers acutely surperfused with amiodarone, but not in fibers of chronically pretreated dogs [158]. A slope decrease in the early phase of ER_S1-S2,DI_ was also found in one-dimensional simulations of ventricular normal and ischemic tissue, numerically reconstructed from the ten Tusscher ventricular AP model [147, 170]. The flattening of ER_dyn,DI_ curve counterbalances in this case the pro-arrhythmic APD prolongation.

Dofetilide prolongs ventricular APD and is frequently associated with increased pro-arrhythmic risk. This has been attributed by Osadchii, in his study on Langendorff-perfused guinea pig hearts, to the action of the drug in steepening the early phase of ER_S1-S2,DI_ curve and increasing its rate constants [109, 110]. In addition, not only dofetilide increases the early part of ER curve, but it does so with a different extent in different regions of the ventricle, accentuating physiological heterogeneities in restitution properties and leading, in turn, to abnormal repolarization gradients, which promote arrhythmias [113].

UK-68,798 is another potent class III antiarrhythmic drug which prolongs APD at the nanomolar concentrations, thus shifting the ER_S1-S2,DI_ curve upwards, without altering, though, its kinetics properties like time constant or S_max_ in dog isolated cardiac Purkinje fibers [77]. In the case of EGIS-7229, class III properties are present at low concentration, whereas at higher concentrations, it mainly shows class I action, like increasing the time constant of ER_S1-S2,DI_ in guinea pig papillary muscle [117]. The steepening of ER_S1-S2,DI_ curve exerted by class III antyarrhythmics has also been investigated in simulation studies by means of numerical AP models [124].

### Class IV

This class of drugs are blockers of calcium channels. Verapamil has been shown to reduce the slope of both ER_S1-S2,DI_ and ER_dyn,DI_ curves in microelectrode impaled dog endocardium (Fig. 18D), which likely explain its ability to prevent the induction of VF [127]. Interestingly, beside flattening ER_S1-S2,DI_ and ER_dyn,DI_ curves, verapamil also reduces the thickness of the hysteretic ER_btb,DI_ and ER_btb,CI_ under oscillatory pacing, as shown by Guzman in isolated ventricular tissue from pigs (Guzman et al. 2010). I have shown the same effect in a simulations study on a human ventricular AP model [183] by selectively reducing I_CaL_ under oscillatory pacing. Both studies emphasize the role of STM in shaping the hysteretic profile of ER_btb_, its relevance in predicting repolarization stability, and its modulation by drugs of this class.

## Conclusions

Two impressions emerge in reviewing the literature on cardiac cellular electrical restitution. The first, positive, is its largely established ability to predict arrhythmic conditions; the second, negative, is that the term restitution is still loosely applied, which causes ambiguities and contradictions in the interpretation of experimental results.

I have forced into this manuscript a heavy notation to indicate the different restitution protocols, as I believe the distinction between the different aspects of restitution dynamics, provided by the different protocols, is a key point in this matter.

As I have mentioned above, the original meaning of restitution concerns the ability to predict how the excitable membrane will respond, in the short- or long-term, to changes in the pacing rate. What electrophysiologists want to know is, for instance, the following: what happens to the APD, or, equivalently, to the refractory period, when a constant or variable pacing CL is suddenly shortened? Is there any pacing condition that makes repolarization more stable? What is the role of a specific ionic current in modulating the APD response? Can cardiac memory be removed? or added? and so on. The answer to these questions can be given by conceiving theoretical constrains to reduce the intrinsic unpredictability of the non-linear dynamic system of cardiac ventricular AP, and translate them into feasible experimental protocols, taking advantage of the growing body of theoretical, computational, and technical solutions that electrophysiology continuously provides. This is the aim of carefully looking at the literature on cardiac cellular electrical restitution, and I do hope this paper will give some contribution to the effort.

## Data Availability

All data presented in this paper are available at the cited references.

## Abbreviations

AP: Action potential
APD: Action potential duration
BCL: Basic cycle length
CL: Pacing cycle length
CI: Coupling interval (same as rest interval RT, test interval, or S2)
EC: Excitation-contraction
ER: Electrical restitution
ER_dyn_: Dynamic electrical restitution
ER_S1-S2_: Standard electrical restitution
ER_…,DI_: Restitution measured as APD vs DI
ER_…,CI_: Restitution measured as APD vs CI
ER_APC_: Restitution of ionic fluxes obtained by AP-clamping the membrane
ER_btb_: Beat-to-beat electrical restitution
ER_CB_: Constant BCL restitution
HRV: Heart rate variability
LCB: Last conditioning beat
MAP: Monophasic action potential
RRD: Reverse rate dependence
RT: Rest interval (same as CI, test interval, or S2)
S1: Conditioning cycle length
S2: Pre-test cycle length (same as CI, rest interval RT, test interval)
S_max_: Maximum value of ER slope over the entire measured curve
VF: Ventricular fibrillation
